# Induction of MET Receptor Tyrosine Kinase Down-regulation through Antibody-mediated Receptor Clustering

**DOI:** 10.1038/s41598-018-36963-3

**Published:** 2019-02-13

**Authors:** Wenjing Li, Adam Dick, Fei Lu, Hui Zhang, Hong Sun

**Affiliations:** 1 School of Chemical Biology and Biotechnology, Peking University Shenzhen Graduate School, Shenzhen, Guangdong, China; 2 Department of Chemistry and Biochemistry, University of Nevada, Las Vegas, Las Vegas, NV 89154-4003 USA

## Abstract

The proto-oncoprotein MET is a receptor tyrosine kinase that plays a key role in cancer cell growth and invasion. We have used fluorescence-tagged antibodies to activate MET in live serum-starved glioblastoma cells and monitor the fate of antibody-bound MET receptor in single cell-based assays. We found that the antibodies induced rapid and transient formation of highly polarized MET clusters on the plasma membrane and promoted the activation of MET, resembling the initial effects of binding to its ligand, HGF. However, the antibody-induced clustering and activation of MET led to the rapid removal of the receptor from cell surface and altered its intracellular processing, resulted in rapid degradation of the receptor. Consequently, while cells pre-treated with HGF remain competent to respond to further HGF stimulation, cells pre-treated with antibodies are refractory to further HGF stimulation due to antibody-mediated MET depletion. Removal of MET by sustained treatment of antibodies blocked cancer cell migration and invasion. Our studies reveal a novel mechanism to alter the recycling process of MET in glioblastoma cancer cells by promoting the receptor degradation through a proteasome-sensitive and lysosome-dependent pathway through the ligand-independent activation of MET using anti-MET antibodies.

## Introduction

The *Met* oncogene was originally identified as a chromosomal translocation fusion gene, which encode the oncogenic TPR-MET fusion protein in a chemically transformed human osteosarcoma-derived cell line^1^. The *TPR-MET* fusion oncogene expresses a constitutively active MET receptor tyrosine kinase (RTK) activity due to the dimerization of the leucine-zipper domain in the TPR (Translocated Promoter Region) moiety of the fusion protein^2^. The MET (also called c-MET) RTK is normally expressed in various cells of epithelial origins or fibroblasts, and is essential for embryonic development, mitogenesis and morphogenesis of various tissues such as skeletal muscle, limb, and neural crest development^3,4^. The MET RTK is activated by the binding of its cognate ligand, hepatocyte growth factor (HGF), which induces the phosphorylaton of two tyrosine residues, tyrosine-1234 and tyrosine-1235 (Y1234/Y1235) of the catalytic loop of the kinase domain^5^. MET activation mobilizes the coordinated invasive cell growth program by promoting cell proliferation, survival, migration, and morphogenesis^3,4^.

Altered expression of MET is associated with various malignancies. Amplification of the *Met* gene is identified in medulloblastoma, gastric and esophageal carcinomas, and non-small-cell lung (NSCL) carcinoma with acquired resistance to epidermal growth factor receptor (EGFR) inhibitor, whereas activating mutations of MET are associated with sporadic papillary renal cancer, childhood hepatocellular carcinoma and gastric carcinoma^6^. The expression of MET is also aberrantly up-regulated in many human malignancies including glioblastoma multiforme (GBM)^7^, the most aggressive and therapeutically difficult brain tumor^8^. In normal cells, HGF-induced MET activation is a tightly regulated process^9^. After ligand binding, MET is internalized via endocytosis and the tyrosine-phosphorylated receptor is recognized by CBL ubiquitin E3 ligase to target MET to multivescular bodies for subsequent degradation in lysosomes^9^. Notably, certain mutations in the kinase domain of MET, originally identified in human renal papillomas, allow the receptor to constitutively recycle back to the cell surface, and these mutations lead to stronger signaling activities^10^. Abnormal activation of MET is responsible for resistance to targeted therapies against VEGFR (vascular endothelial growth factor receptor) in GBM^11,12^ and inhibitors of the EGFR in lung cancers^13,14^. Over-expression or ligand-mediated activation of the MET signaling pathway is an established mechanism of resistance towards the targeted therapies against members of EGFR subfamily of RTKs^6^.

Since the high level expression of MET is correlated with poor prognosis of various cancers, MET serves as an excellent target for cancer therapy. Various approaches, such as the development of small molecular chemical inhibitors or specific monoclonal antibodies, have been explored to inhibit the RTK activity of MET or to block the interaction between the MET receptor and the ligand, HGF, in a wide array of cancers^15,16^. An one-armed monovalent 5D5 antibody has been developed^17–19^ that binds to the monomeric MET protein on the cell surface and blocks the binding of HGF to the receptor without induction of the down-regulation of the MET receptors. A non-activating monoclonal antibody, LY2875358, was recently reported^20^. This antibody can prevent the MET receptor to interact with HGF, as well as to trigger receptor downregulation^20^. Another bivalent antibody, SAIT301, which does not activate the RTK activity of MET, was also shown to cause the downregulation of the MET protein after an extended treatment^21^. It appears that LY2875358 and SAIT301 employ different cellular processes to down-regulate MET receptors, although a direct comparison of these two antibodies is lacking. These studies suggest that the MET receptor, with its unique structural or conformational determinants, may be manipulated through binding with antibodies to target the receptors to degradation.

We have recently found that the MET receptor is often complexed with AXL, another important RTK, in glioblastoma and breast cancer cells^22^. HGF stimulation induces an enhanced formation of the MET-AXL complex to promote the MET-AXL co-clustering on the plasma membrane^22^. Our findings reveal that HGF-dependent clustering of the MET-AXL complex is required for the MET-mediated cell signaling. In addition, we have recently shown that the cell surface delivery of MET from Golgi is a highly regulated process which is very sensitive to the lipid modification enzyme ASM (acid sphingomyelinase) in GBM cancer cells, suggesting that the total level of cell surface MET is closely regulated by both newly synthesized MET from Golgi and recycling pool of MET^23^. In this report, we have examined the cellular response to the ligand-independent activation of MET receptor on the plasma membrane by antibody-mediated MET receptor clustering.

## Results

### Clustering of MET on the plasma membrane by APC-conjugated anti-MET antibodies

**Figure 1:**
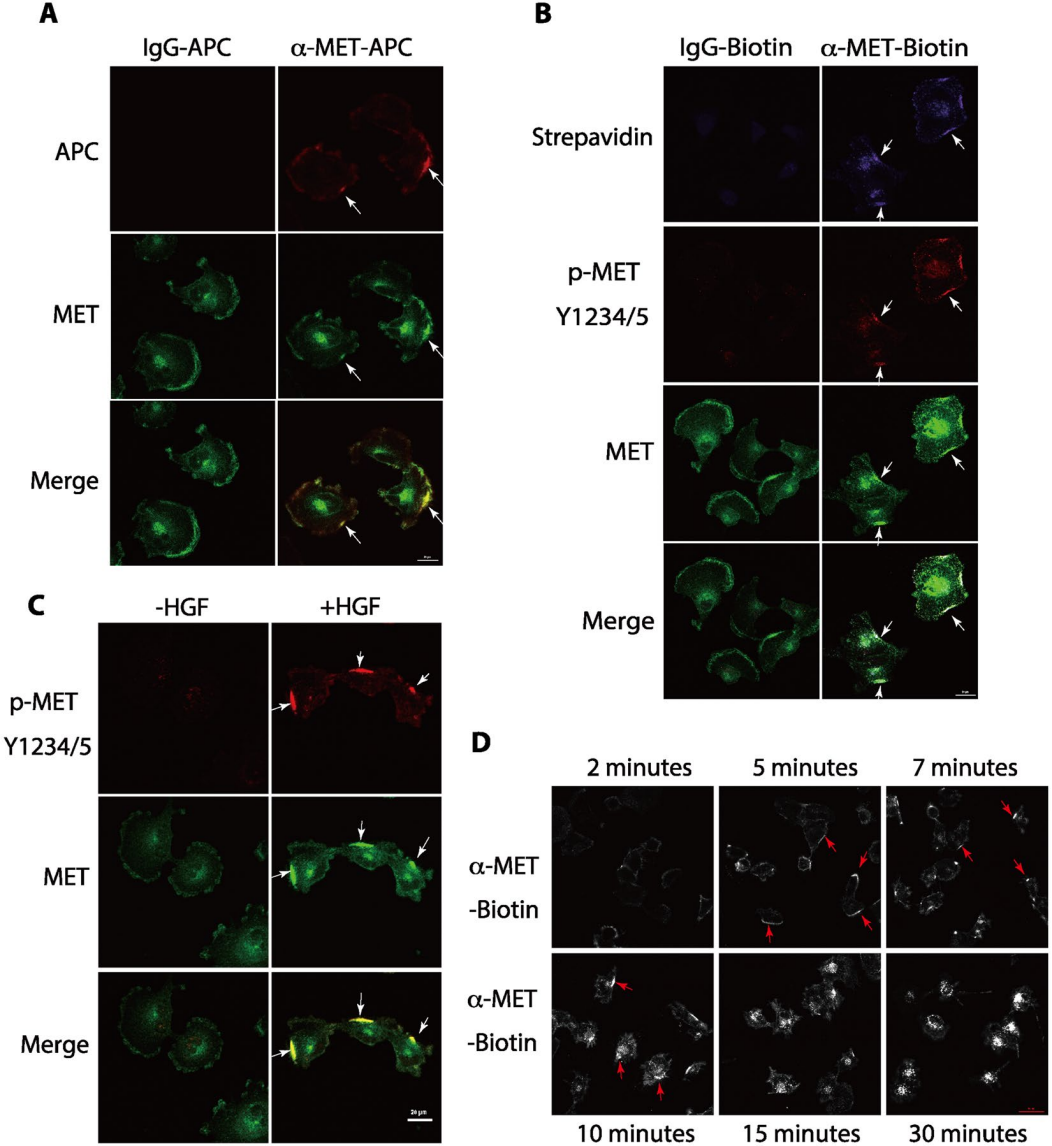
Antibody-mediated MET receptor crosslinking leads to the receptor clustering and patch formation on the plasma membrane through activation of MET receptor. (**A**) Live U373-MG cells, which were serum starved for three hours, were incubated with the APC-conjugated mouse monoclonal anti-MET antibody (1 μg/ml) (red) or its isotype control, APC-conjugated, mouse IgG (1 μg/ml) for 7 minutes. Cells were then washed, fixed and co-immunostained with an anti-MET antibody pre-conjuagted to Alexa Fluor 488, which detects the total MET protein (green). Cells were imaged by confocal microscopy. Clusters (or patches) of the MET proteins on the plasma membrane are indicated by arrows. (**B**) Live serum-starved U373-MG cells were treated with biotin-conjugated goat anti-MET antibodies (2 μg/ml) or biotin-conjugated normal goat IgG antibodies (2 μg/ml) (control) for 7 minutes. Cells were then washed, fixed and immunostained with the Streptavidin-conjugated to Brilliant Violet 421 (BV421) to detect the biotin-conjugated antibodies (purple), a rabbit anti-Y1234/1235-phosphorylated MET antibody for the activated MET protein (secondary antibody conjugated with Alexa Fluor 647, red), and an anti-MET antibody pre-conjugated to Alexa Fluor 488 for total MET protein (green). (**C**) Serum-starved cells were left untreated or stimulated with HGF (50 ng/ml) for 7 minutes. Cells were fixed and immunostained with a rabbit antibody against Y1234/Y1235-phosphorylated MET for the activated MET protein (red) and with an anti-MET antibody pre-conjugated with the Alexa Fluor 488 to detect total MET (green). (**D**) Serum-starved cells were treated with the biotin-conjugated anti-MET antibody (2 μg/ml) and fixed at the various time points as indicated. Cells were immunostained with strepavidin-conjugated to BV421 as in panel B. Scale bars in (**A–C**), 20 μm.

Our recent studies showed that the presence of MET RTK on the plasma membrane is dynamically regulated by ceramides generated by ASM^23^ and HGF promotes the MET-AXL RTK complex formation on the plasma membrane^22^. To examine how MET is activated on the plasma membrane, we examined the distribution of the MET protein in serum-starved human U373-MG glioblastoma cells, which express relatively high levels of MET protein^23^. To monitor the distribution of MET on the plasma membrane, we exposed live serum-starved cells to mouse monoclonal anti-MET antibodies that were pre-conjugated with Allophycocyanin (APC), an intensely bright phycobiliprotein that exhibits far-red fluorescence with high quantum yields. The advantage of using the APC-conjugated MET antibody to immunostain live cells is that it only detected the plasma membrane-associated MET with high signal-to-noise ratio (Fig. 1A, top row). Interestingly, we found that addition of the APC-tagged mouse monoclonal anti-MET antibody to the live serum-starved cells specifically detected MET proteins in a highly “clustered” pattern on the plasma membrane in the live and serum-starved cells, while the control APC-tagged mouse IgG did not produce any fluorescence signals in these cells (Fig. 1A, top panels).

We also monitored the distribution of total MET proteins after fixation and permeabilization of cells, using rabbit anti-MET antibodies which are pre-conjugated to the Alexa Fluor 488 fluorophore, in a regular immunostaining procedure (Fig. 1A, middle row). The anti-MET antibodies (detected by Alexa Fluor 488 fluorescence) immunostaining revealed that MET is weakly and evenly distributed on the plasma membrane in the serum-starved cells in the cells that exposed to the APC-IgG control antibody (Fig. 1A). The MET staining was also present in the intracellular compartments. However, addition of the APC-conjugated anti-MET antibodies appeared to cause a re-distribution of the MET protein on the plasma membrane that showed more polarized and “clustered” immunostaining patterns of the receptors (Fig. 1A, middle row). The clustered areas of MET overlapped with that of the area occupied by the APC-conjugated MET antibodies on the plasma membrane (Fig. 1A, bottom row), raising the possibility that the clustering of the MET protein on the plasma membrane is caused by the APC-conjugated anti-MET antibodies.

### Clustering and activation of MET on the plasma membrane by biotin-conjugated anti-MET antibodies

The clustering of the MET protein on the plasma membrane in response to the APC-conjugated MET antibody is reminiscent of T cell receptor activation by bivalent antibodies. T cell receptors have been shown to be activated by the binding of bivalent antibodies to promote the oligomerization of the receptors and subsequent assembly of multi-protein complexes, leading to the formation of a “patch” (also referred to as “cap” or “cluster”) of the receptor complexes on the T cell membrane^24^. We have recently found that in various cancer cells, MET can be induced to form clusters on the plasma membrane in response to its natural ligand HGF stimulation^22^. It is known that when MET receptors are bound by their cognate ligand HGF, receptors are induced to form dimers and the kinase activity of MET is turned on by trans-phosphorylation of Y1234 and Y1235 within the transactivation loop^5^. Since the addition of APC-conjugated anti-MET antibody to the serum-starved cells showed the clustering of MET on the plasma membrane, we wondered whether clustering of the MET receptor on the plasma membrane is accompanied by receptor dimerization and ligand-independent activation of the MET RTK activity.

To test whether antibody-mediated cross-link of the receptor is sufficient to activate MET, we used an independent bivalent goat anti-MET antibody that was pre-conjugated to biotin. The conjugation of antibodies to biotin allows the subsequent detection of the receptors by Streptavidin-labeled Brilliant Violet 421. Since the excitation of Brilliant Violet 421 by 405 nm laser induces a fluorescence maximum emission of 421 nm, it is possible to use three spectrum channels to simultaneously monitor and compare the distributions of biotinylated antibodies (purple), the activated MET protein using a rabbit anti-phosphorylated Y1234/1235 antibody followed by a secondary antibody conjugated with the Alexa Fluor 647 fluorophore (red), and subsequent detection of the total MET proteins using an anti-MET antibody pre-conjugated to Alexa Fluor 488 (green). Our studies showed that exposure of live serum-starved cells to the biotinylated goat anti-MET antibody, but not a control biotinylated normal goat IgG, was sufficient to induce the clustering and patch formation of the MET proteins on the plasma membrane (Figs 1B, S1A). These clustered MET areas represent the activated form of MET proteins since they are phosphorylated at Y1234/Y1235, a pair of tyrosine residues that are autophosphorylated by the activated MET RTK. Our studies indicate that the RTK activity of MET can be activated by receptor clustering through the binding of the bivalent antibodies to the MET receptor on the plasma membrane in live serum-starved cells, and such events can be detected at a single cell level.

In addition, the biotin-conjugated goat anti-MET antibodies also induced the cluster formation of the phosphorylated MET receptor on the plasma membrane in other human glioblastoma cells such as T98G cells (Fig. S1B), suggesting the ability to induce the activation and cluster formation by the biotinylated anti-MET antibodies are not limited to U373-MG cells.

### Clustering of MET on the plasma membrane by ligand binding

The induction of clustered or patched MET distribution on the plasma membrane by bivalent antibodies suggests that the MET RTK on the plasma membrane may be activated through the similar manner by its natural ligand, HGF. Indeed, we have recently found that addition of HGF to the serum-starved cells caused a rapid and dramatic change of the MET distribution on the plasma membrane (Fig. 1C)^22^. We tested the effects of HGF at 50 ng/ml, the lowest concentration of HGF needed to maximally activate MET in U373-MG cells^22^, and the concentration is also in the range of HGF known to be present in the tumor microenvironment in the melanoma samples carrying *BRAF* mutations^25^. In serum-starved cells, there was no immunostaining signal when stained with the anti-phosphorylated Y1234/Y1235 antibodies on the plasma membrane, suggest that MET is not activated (Fig. 1C). Addition of HGF to the serum-starved cells induced a strong staining from antibodies specific for the Y1234/Y1235-phosphorylated MET, which also exhibited the highly polarized and clustered phospho-MET staining patches that strongly overlapped with the staining with anti-MET antibodies (conjugated to Alexa Fluor 488) on the same cell (Fig. 1C). Thus, the clustered staining of MET or phospho-MET represents the presence of the active MET forms on the plasma membrane, which represent early events detectable at the single cell level during HGF-induced MET receptor activation (Fig. 1C)^22^.

### The clustering of the MET protein by the biotin-conjugated anti-MET antibodies occurred rapidly and transiently on the plasma membrane

To determine the temporal response of the MET proteins towards specific antibodies, we monitored the cluster formation of the receptor on the plasma membrane at various times after addition of the biotin-conjugated anti-MET antibodies to the serum-starved cells (Fig. 1D). Our studies revealed that the antibody-activated clustering of the MET protein on the plasma membrane occurred very rapidly and transiently (Fig. 1D). Addition of the biotin-conjugated anti-MET antibody between 2 to 5 minutes caused the patched clustering of the MET protein on plasma membrane in each cell (Fig. 1D). The MET staining became a single bright spot or a strong patched area on the surface of each cell between 7–10 minutes after exposure to the biotin-conjugated anti-MET antibody, with some cells started to show internalized biotin-conjugated anti-MET antibody at 10 minutes. Within 15–30 minutes, the MET staining disappeared from cell surface, with concurrent strong intracellular staining inside of the cell, suggesting the internalization of the antibody-MET complexes. These observations revealed that the antibody-mediated clustering of MET induced the receptor activation and the activated receptors are quickly internalized, independent of the ligand binding.

### Both APC- or biotin-conjugated anti-MET antibodies transiently activate the MET RTK signaling process

**Figure 2:**
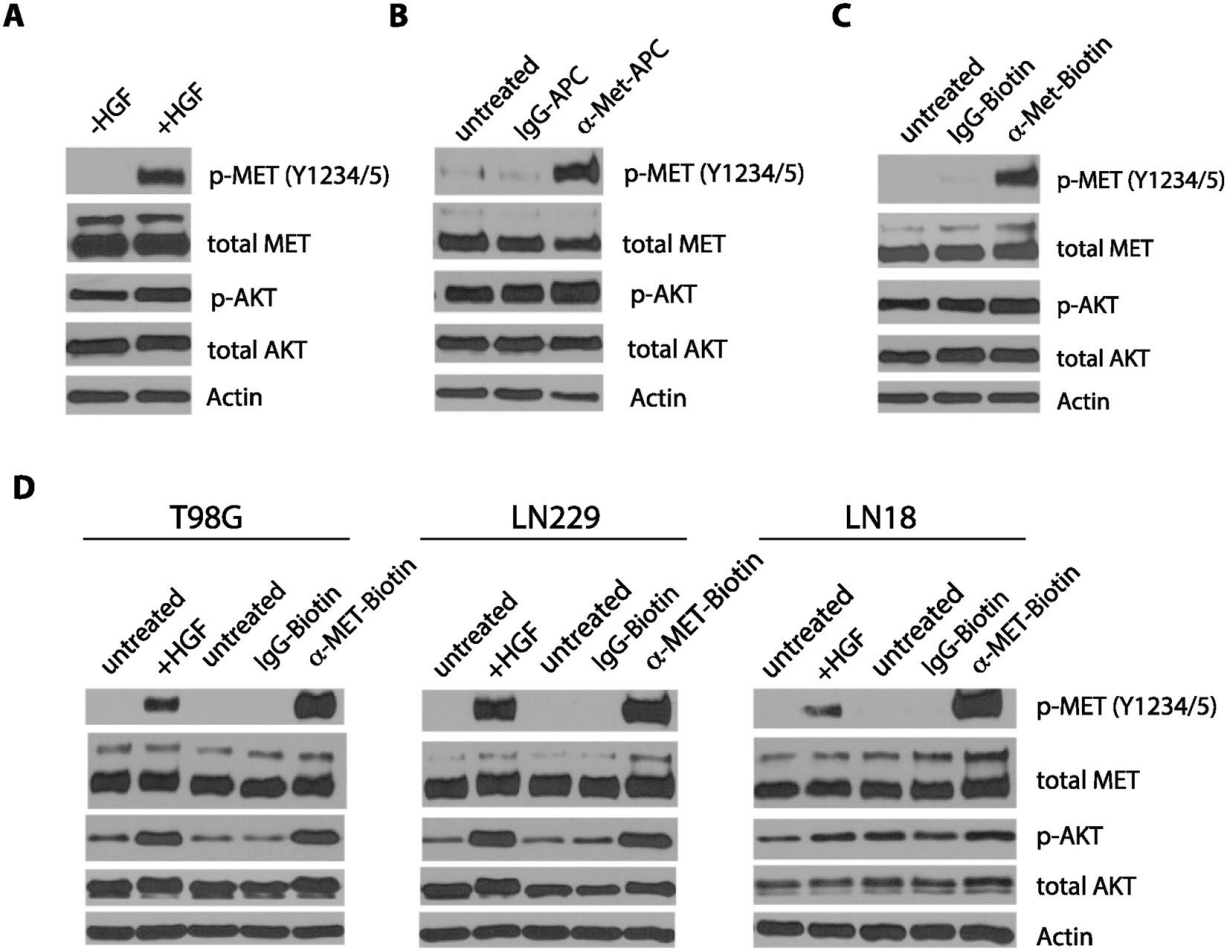
The MET receptor is activated by HGF, or APC- or Biotin-conjugated anti-MET antibodies. (**A**) Serum-starved U373-MG cells were treated either with HGF (50 ng/ml) for 7 minutes or left untreated (-HGF) as indicated. The cells were directly lysed in SDS buffer and proteins were detected by Western blotting with antibodies for the Y1234/Y1235-phosphorylated MET (p-MET, Y1234/5), total MET, phosphorylated AKT (p-AKT), total AKT, or actin (loading control), as indicated. (**B**) Serum-starved U373-MG cells were left untreated, or treated with APC-conjugated mouse IgG control antibody or APC-conjugated mouse monoclonal anti-MET antibody (each at 1 μg/ml) for 7 minutes. Cells were directly lysed in SDS buffer and proteins were detected by Western blotting with antibodies as in panel A. (**C**) The same as panel B except that the Biotin-conjugated goat control IgG or Biotin-conjugated goat anti-MET antibodies (each at 2 μg/ml) were used. (**D**) Serum-starved human glioblastoma cells T98G, LN229, and LN18 cells were treated with HGF or biotin-conjugated IgG or biotin-conjugated anti-MET antibodies as in panel A and C, or left untreated. The proteins were detected by Western blotting with antibodies as in panel A.

We have further biochemically examined and compared the ligand- and antibody-induced MET activation. In serum-starved cells, MET was not phosphorylated on Y1234/Y1235 (p-Y1234/Y1235), as revealed by Western blotting analysis. Addition of HGF to the serum-starved cells promoted the phosphorylation of Y1234/Y1235 on MET within 7 minutes (Fig. 2A). In addition, HGF initiated a cascade of MET signaling events, including the phosphorylation and activation of downstream kinases such as AKT (Fig. 2A). We found that addition of the APC- and biotin-conjugated anti-MET antibodies, but not the control normal IgG which were similarly conjugated to APC or biotin, to the live serum-starved cells, each induced the phosphorylation of Y1234/Y1235 on MET within 7 minutes (Fig. 2B,C). To determine whether the activation of MET by these conjugated antibodies is not limited to human U373-MG glioblastoma cells, we also tested the response of other human glioblastoma cell lines, such as T98G, LN229, and LN18 (Fig. 2D). We found that APC- and biotin-conjugated anti-MET antibodies strongly induced MET and AKT phosphorylation, as compared to the HGF control, in all these glioblastoma cell lines (Fig. 2D). These observations suggest that the anti-MET antibodies can effectively activate MET in a variety of glioblastoma cell lines.

### Activation of the MET signaling and clustering by APC- or biotin-conjugated anti-MET antibodies requires the RTK activity of MET

**Figure 3:**
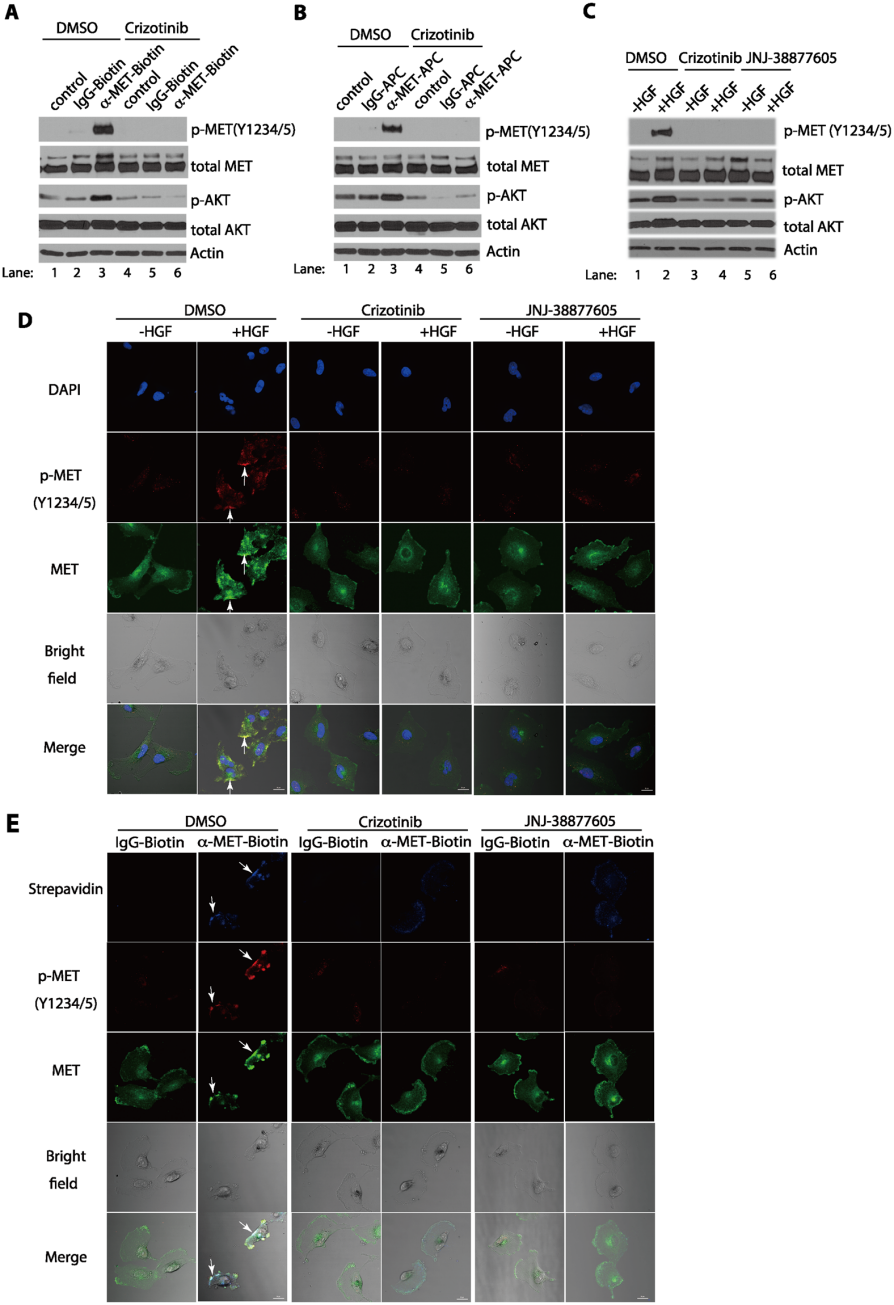
Activation of the MET receptor by HGF or anti-MET antibodies requires the MET RTK activity. (**A**) Serum-starved U373-MG cells were treated with the MET kinase inhibitor, crizotinib (1 μM) (lane 4–6) or DMSO (lane 1–3) for 3 hours. The cells were then treated either with biotin-conjugated anti-MET antibodies or biotin-conjugated IgG (each at 2 μg/ml) for 7 minutes, or left untreated, as indicated. The cells were directly lysed in SDS buffer and proteins were detected by Western blotting with antibodies for the Y1234/Y1235-phosphorylated MET (p-MET, Y1234/5), total MET, phosphorylated AKT (p-AKT), total AKT, and actin (loading control), respectively, as in Fig. 2A. (**B**) The same as in panel A except that the APC-conjugated mouse monoclonal anti-MET antibodies or mouse IgG control antibodies (each at 1 μg/ml) were used. (**C**) Serum-starved U373-MG cells were treated with dimethyl sulfoxide (DMSO) vehicle control (lane 1, 2), or with the MET kinase inhibitors crizotinib (1 μM) (lane 3, 4) or JNJ-38877605 (1 μM) (lane 5,6) for 3 hours. Cells were then treated with or without HGF (50 ng/ml) for 7 minutes as indicated. The cells were directly lysed in SDS buffer and proteins were detected by Western blotting with indicated antibodies. (**D**) Serum-starved U373-MG cells were treated with the MET kinase inhibitors, crizotinib, JNJ-38877605 or DMSO as in panel C. Cells were stimulated with or without HGF (50 ng/ml) for 7 minutes and were subsequently fixed and stained with rabbit antibody against Y1234/Y1235-phosphorylated MET (red) for the activated MET protein and with the Alexa Fluor 488 pre-conjugated anti-MET antibody for total MET protein (green), and bright field for the contrast phase. Cells were also counter stained with DAPI for nuclei. MET receptor clusters (patches) are indicated by arrows. (**E**) Serum-starved U373-MG cells were treated with the MET inhibitors crizotinib, JNJ-38877605 or dimethyl sulfoxide (DMSO) as in panel C. The live serum-starved U373-MG cells were then treated with biotin-conjugated goat anti-MET antibodies (2 μg/ml) or biotin-conjugated goat IgG antibodies (2 μg/ml) (control) for 7 minutes. The cells were then fixed and stained with streptavidin (labeled with Brilliant Violet 421) for biotin-conjugated antibodies (purple) and the rabbit anti-Y1234/1235-phosphorylated MET antibodies (followed by secondary antibodies conjugated to the Alexa Fluor 647) for the activated MET protein (red), and with the Alexa Fluor 488 pre-conjugated anti-MET antibody for total MET protein (green). MET receptor clusters (patches) are indicated by arrows. Scale bars in (**D,E**), 20 μm.

The activation of MET by the addition of the APC- or biotin-conjugated anti-MET antibodies in serum-starved cells represents a ligand-independent activation of the MET receptor on the plasma membrane. The binding of these conjugated antibodies to MET is likely to stimulate the cross-phosphorylation of the MET receptor proteins on the plasma membrane, which is reminiscent of its natural ligand, HGF, binding to the receptor. To verify that the activation of MET by these conjugated antibodies is dependent on the activity of the MET receptor, we used crizotinib^26^, a relative specific tyrosine kinase inhibitor (TKI) of MET. Our studies showed that inhibition of the MET RTK activity by crizotinib indeed effectively blocked the ligand-independent MET activation and MET signaling to AKT by the biotin-conjugated or APC-conjugated anti-MET antibodies, in a manner similar to the crizotinib’s effect on the HGF-dependent activation process (Fig. 3A–C).

The clustering of the phosphorylated MET protein on the plasma membrane by the APC- or biotin-conjugated anti-MET antibodies in the serum-starved cells is likely due to the actions of the activated MET proteins on the plasma membrane (Fig. 1). We, therefore, also examined whether the formation of the MET clusters after the addition of the APC- or biotin-conjugated anti-MET antibodies required the kinase activity of MET RTK. Our studies revealed that the formation of clustered and activated MET proteins on the plasma membrane was abolished by the pre-treatment of cells with crizotinib in U373-MG cells, using cells treated with HGF as controls (Figs 3D,E; S2A). Although crizotinib is often used as an inhibitor for MET^25^, the chemical is also known to inhibit ALK or ROS-1 RTKs^26^. We therefore further tested the effects of other MET inhibitors, JNJ-38877605^27^ or SGX-523^28^, which are more selective MET TKIs than crizotinib. Our data showed that the MET activation and clustering induced by HGF or by anti-MET antibodies was each potently inhibited by JNJ-38877605 (Fig. 3C–E) or SGX-523 (Fig. S2B,C), suggesting that the kinase activity of MET was involved in the antibody-mediated receptor clustering process. Together, our results indicate that the bivalent anti-MET antibodies can activate MET RTK which in turn triggers the receptor clustering, in a manner similar to that of HGF binding to the MET receptor.

### The pre-treatment of cells with biotin-conjugated anti-MET antibodies abolishes the response to HGF

**Figure 4:**
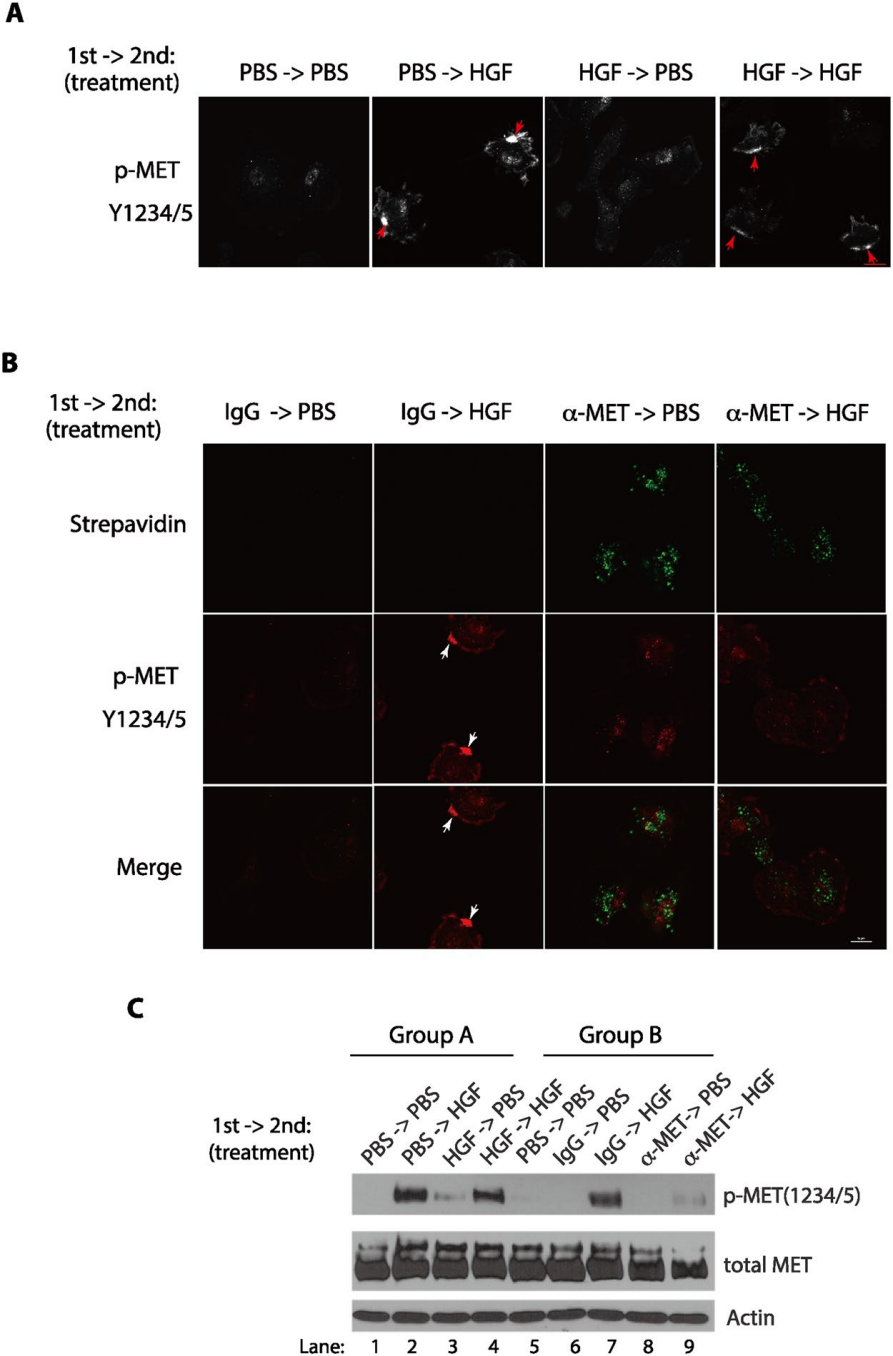
Pre-treatment of the serum-starved cells with the biotin-conjugated anti-MET antibodies renders the cells refractory to the subsequent HGF stimulation. (**A**) U373-MG cells were serum-starved for 3 hours. Cells were pulse-treated (1^st^ treatment) with PBS (phosphate buffered saline, control) or HGF (50 ng/ml) for 7 minutes, washed extensively with warm PBS buffer, and then incubated in the serum-starvation medium for 30 minutes to recover. They were then re-stimulated (2^nd^ treatment) with HGF or PBS (as control) for another 7 minutes. Cells were fixed and immunostained with the anti-Y1234/1235-phosphorylated MET antibodies. MET receptor clusters (patches) are indicated by arrows. (**B**) Serum-starved U373-MG cells were pulse-treated (1^st^ treatment) with the Biotin-conjugated anti-MET antibodies or biotin-conjugated IgG (each at 2 μg/ml) for 7 minutes, washed extensively with warm PBS buffer, and then incubated in the serum-starvation medium for 30 minutes to recover. Cells were then stimulated (2^nd^ treatment) with HGF (50 ng/ml) or PBS (control) for another 7 minutes. Cells were fixed and immunostained with streptavidin (pre-labeled with Brilliant Violet 421, green) and anti-Y1234/1235-phosphorylated MET antibodies (secondary antibody conjugated with Alexa Fluor 647, red). MET receptor clusters (patches) are indicated by arrows. Scale bar in B, 20 μm. (**C**) Serum-starved U373-MG cells were treated as in (**A,B**), with the 1^st^ treatment being either HGF (50 ng/ml) or PBS control (lanes 1–5) or alternatively with the biotin-conjugated anti-MET antibodies or the control IgG (each at 2 μg/ml) (lanes 6–9), for 7 minutes, as indicated. Cells were washed extensively with warm PBS and then recovered in the serum-starvation medium for 30 minutes. Cells were subsequently stimulated (2^nd^ treatment) with HGF (50 ng/ml) or PBS (control) for another 7 minutes, as indicated. Cells were lysed and the levels of the Y1234/1235-phosphorylated MET and total MET proteins were analyzed by Western blotting with specific antibodies as indicated.

Our analysis of the time course of the MET protein have shown that addition of the biotin-conjugated anti-MET antibodies led to the disappearance of the MET protein on the cell surface, following its transient activation on the plasma membrane (Fig. 1B,D). We designed a series of experiments to examine the fate of the receptors that are bound by the anti-MET antibodies, by testing if cells could still respond to HGF after pre-treatment with the biotin-conjugated anti-MET antibodies. In our control experiments, we pre-treated the serum-starved U373-MG cells with HGF for 7 minutes (1^st^ treatment), which induced the MET activation and receptor clustering on the plasma membrane (Fig. 4A). We next removed the unbound HGF by washing cells with the PBS buffer and re-incubated the cells in the serum-starvation medium for 30 minutes (recovery time) to allow the endocytosis and removal of the activated MET proteins on the cell surface during the 1^st^ HGF treatment. We then re-added HGF the second time for 7 minutes (2^nd^ treatment) to the cells that have been received the 1^st^ HGF treatment, and examined the response of these cells to form the MET clusters (Fig. 4A,C). Our studies revealed that the 2^nd^ HGF addition can still stimulate the cluster formation of the phosphorylated and activated MET proteins in the plasma membrane in cells received the 1^st^ HGF treatment (Fig. 4A). Our Western blotting analysis also revealed that the 2^nd^ HGF addition promotes the similar levels of the Y1234/Y1235-phosphorylated MET protein in the cells that have received the 1^st^ HGF treatment (Fig. 4C), suggesting that the prior HGF treatment did not abolish the responsiveness of the serum-starved cells to the second HGF stimulation.

However, there is a striking difference in the response to the HGF treatment (2^nd^ treatment) if the cells had received the prior or 1^st^ treatment of the biotin-conjugated anti-MET antibodies (Fig. 4B,C). In these experiments, we similarly pre-treated serum-starved cells with the biotin-conjugated anti-MET antibodies for 7 minutes (1^st^ treatment) and removed the unbound antibodies by extensive washing. The antibody-pretreated cells were next incubated in the serum-starvation medium for 30 minutes (recovery time), which greatly eliminated the activated MET protein on the cell surface (Fig. 4B). Cells were then exposed to HGF (2^nd^ treatment) and the cluster formation of the activated and phosphorylated MET protein was monitored. We found that pre-treatment of cells with the biotin-conjugated anti-MET antibodies greatly dampened the responsiveness of the cells to form clusters when exposed to HGF (Fig. 4B). Unlike the 1^st^ treatment of HGF, we found that 1^st^ treatment of the biotin-conjugated anti-MET antibodies greatly reduced the response of cells to HGF (2^nd^ treatment) to activate MET phosphorylation, as revealed by Western blot analysis (Fig. 4C). We also monitored the fate of the anti-MET antibodies after a 7-minute exposure to cells (1^st^ treatment), and found that the antibody-marked MET proteins were accumulated in the lysosomes following a 30-minute chase (Fig. S3). These studies indicate that the biotin-conjugated anti-MET antibodies pre-treatment caused the MET proteins to be routed to the lysosomes, consequently deplete the cell surface MET and prevent cells to further respond to the HGF stimulation.

### Pretreatment of biotin-conjugated anti-MET antibodies blocked subsequent AXL phosphorylation

**Figure 5:**
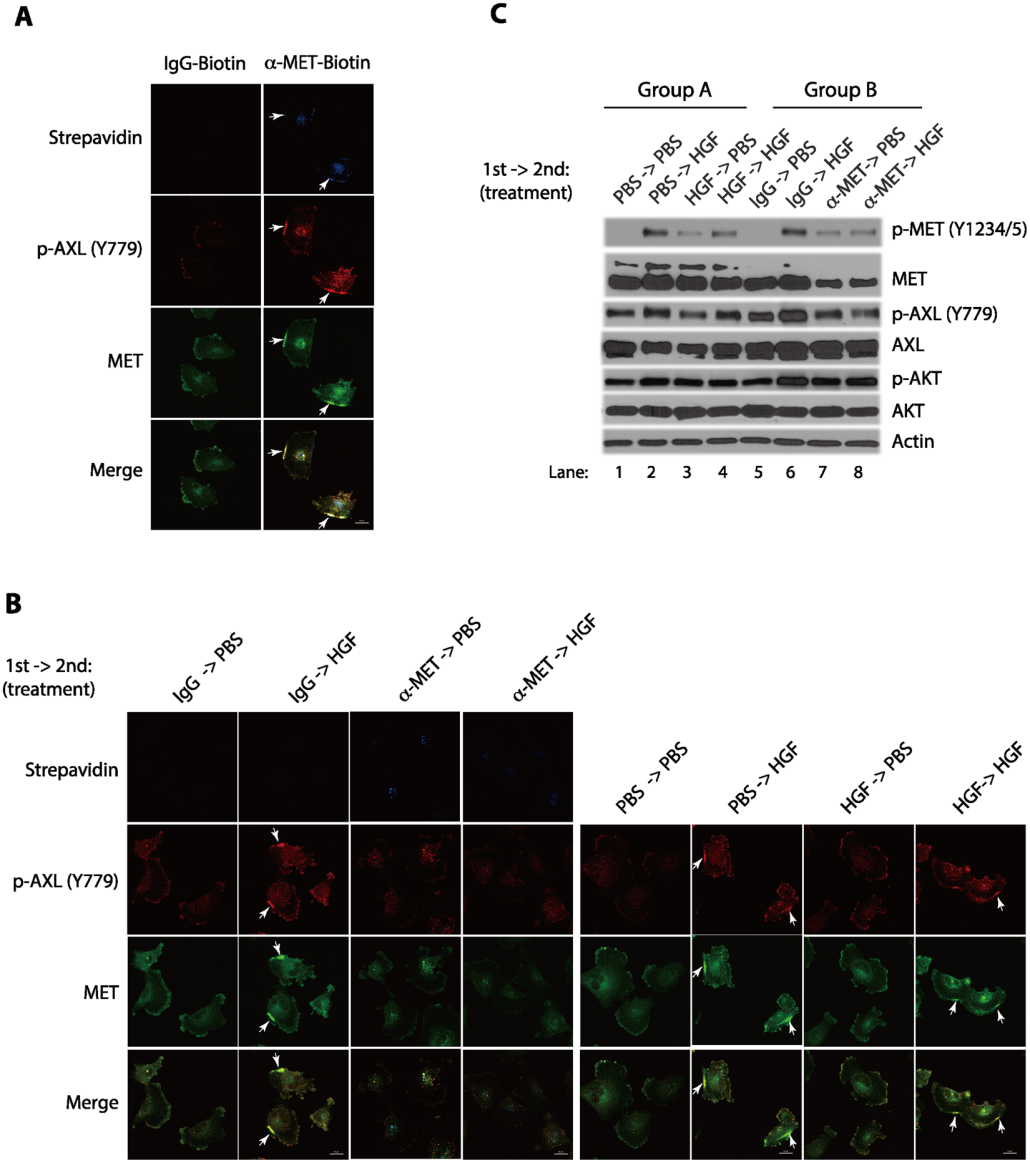
Pre-treatment of the serum-starved cells with the biotin-conjugated anti-MET antibodies caused the activation of AXL refractory to the subsequent HGF stimulation. (**A**) Live serum-starved U373-MG cells were treated with biotin-conjugated goat anti-MET antibodies or biotin-conjugated control IgG (each at 2 μg/ml) for 7 minutes. Cells were then washed, fixed and immunostained with the streptavidin-conjugated to Brilliant Violet 421 to detect the biotin-conjugated antibodies (purple), a rabbit anti-Y779-phosphorylated AXL antibody for the activated AXL protein (secondary antibody conjugated with Alexa Fluor 647, red), and an anti-MET antibody pre-conjugated to Alexa Fluor 488 for total MET protein (green). (**B**) U373-MG cells were serum-starved for 3 hours. Cells were pulse-treated (1^st^ treatment) with the biotin-conjugated anti-MET antibodies or control IgG (each at 2 μg/ml), PBS (control) or HGF (50 ng/ml) for 7 minutes, washed extensively with warm PBS buffer, and then incubated in the serum-starvation medium for 30 minutes to recover. They were then re-stimulated (2^nd^ treatment) with HGF (50 ng/ml) or PBS (as control) for another 7 minutes. Cells were fixed and immunostained with the anti-Y779-phosphorylated AXL antibody or with an anti-MET antibody. MET receptor clusters (patches) are indicated by arrows. Scale bars in (**A,B**), 20 μm. (**C**) Serum-starved U373-MG cells were treated as in B, with the 1^st^ treatment being either HGF or PBS control (lanes 1–4) or alternatively with the biotin-conjugated anti-MET antibodies (2 μg/ml) (lane 7, 8) or control IgG control (2 μg/ml) (lanes 5, 6), for 7 minutes, as indicated. Cells were washed extensively with warm PBS and then recovered in the serum-starvation medium for 30 minutes. Cells were subsequently stimulated (2^nd^ treatment) with HGF (50 ng/ml) or PBS (control) for another 7 minutes, as indicated. Cells were lysed and the levels of the Y1234/1235-phosphorylated MET and total MET proteins, anti-Y779-phosphorylated AXL antibody, total AXL, phosphorylated AKT (p-AKT), total AKT, were analyzed by Western blotting with specific antibodies as indicated.

We have recently found that HGF-induced MET clustering involves a complex formation of MET and AXL, another important RTK in glioblastoma^22^. The co-clustering of MET and AXL is accompanied by AXL phosphorylation at Tyr-779 and AXL activation^22^. We wondered if the anti-MET antibodies induced MET clustering also involves AXL. We found that treatment of cells with the biotin-conjugated antibodies induced the phosphorylation of Tyr-779 in AXL and the phosphorylated AXL was co-clustered with MET (Figs 5A; S4A). Similar events were also observed when cells were exposed to APC-conjugated anti-MET antibodies (Fig. S4B). These observations suggest that the anti-MET antibodies induced the early MET signaling events similar to that of HGF. However, when we performed a sequential HGF treatment experiments, the anti-MET antibodies pretreated cells behaved very differently from the HGF-pretreated cells (Fig. 5B). While cells pretreated with HGF could again induce AXL Tyr-779 phosphorylation and AXL co-clustering, the cells pretreated with anti-MET antibodies failed to respond to the subsequent 2^nd^ treatment of HGF and could no longer induce AXL-MET co-clustering and AXL phosphorylation (Figs 5B; S4C). Western blot analysis confirmed that pre-treatment with anti-MET antibodies blunted the subsequent HGF-induced AXL phosphorylation at Tyr-779, while control cells pretreated with HGF responded to subsequent HGF addition and effectively induced AXL phosphorylation (Fig. 5C). These experiments demonstrate that pre-treatment of anti-MET antibodies can effectively block the subsequent HGF-mediated AXL co-activation in these cancer cells, and such effects can be detected within less an hour following a 7-minute antibody exposure.

### The biotin-conjugated anti-MET antibodies trigger MET proteins degradation

**Figure 6:**
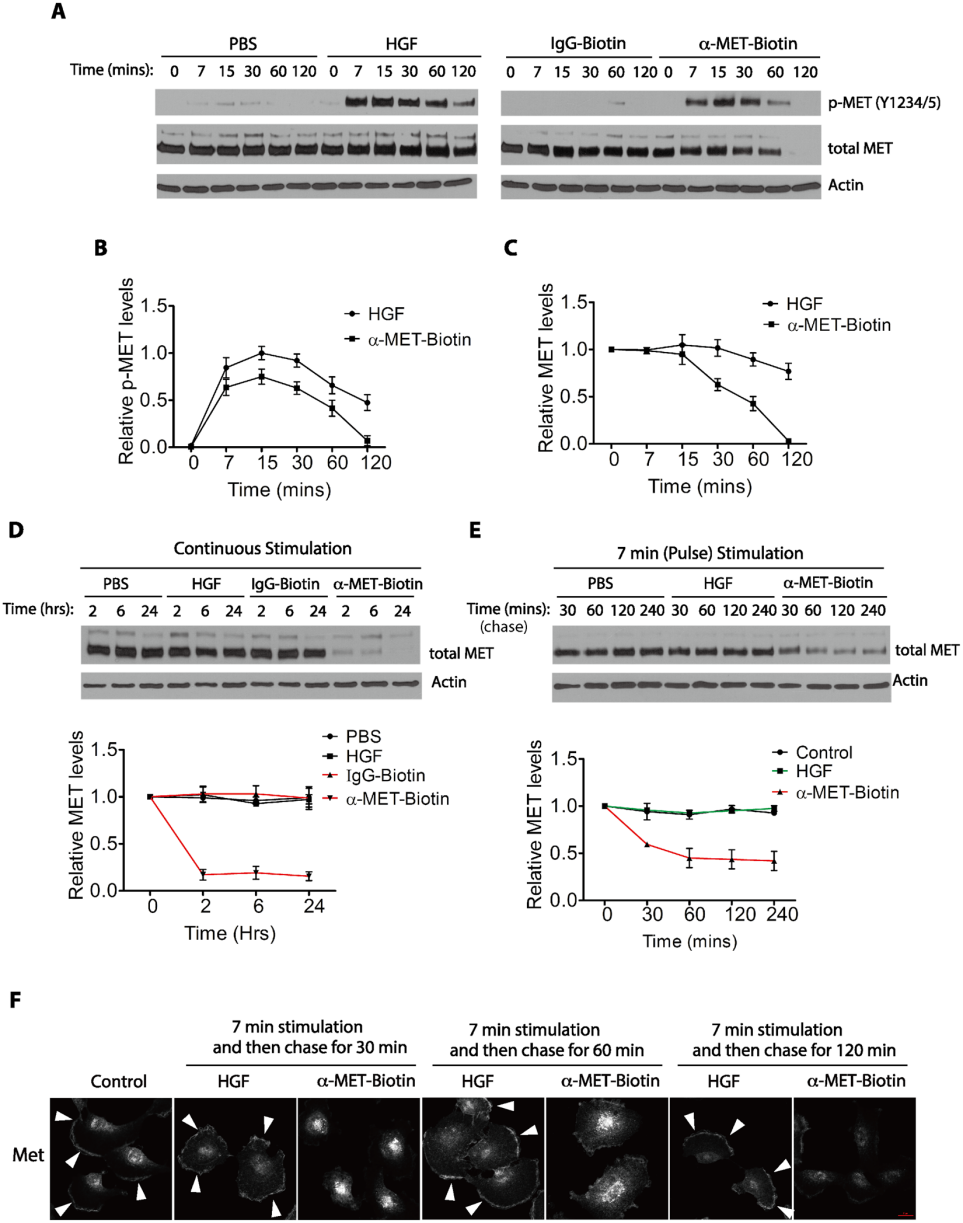
Ligand-independent activation of MET by the Biotin-conjugated anti-MET antibodies promotes degradation of the MET protein. (**A**) Serum-starved U373-MG cells were treated either with HGF (50 ng/ml) or PBS (control), alternatively, with the biotin-conjugated anti-MET antibodies or control IgG (each at 2 μg/ml) for various time points as indicated. The cells were directly lysed in SDS buffer and the protein levels of Y1234/Y1235-phosphorylated MET (p-MET, Y1234/5) and total MET proteins were detected by Western blotting with respective antibodies. Actin is used as the loading control. Shown is a representative set of blots from three independent repeats. (**B**) A quantification of p-MET protein levels in cells treated with HGF or with anti-MET antibodies using data from panel A. The relative p-MET protein levels were first normalized to the actin as loading control, and then calculated using the p-MET levels at 15 min after HGF treatment as the reference point (1.0). Data of quantitation are shown, with the mean and standard deviation (S.D.) for error bars derived from three independent repeats. (**C**) A quantification of total MET protein levels in cells treated with HGF or anti-MET antibodies using data from panel A. The relative total MET protein levels were first normalized to the actin as loading control, and calculated using the MET protein levels at the 0 min time points under each condition as the reference point (1.0). Data of quantitation are shown, with the mean and standard deviation (S.D.) for error bars derived from three independent repeats. (**D**) Serum-starved U373-MG cells were treated either with HGF (50 ng/ml) or the biotin-conjugated anti-MET antibodies (2 μg/ml), or with corresponding PBS and the normal IgG controls, for extended time points as indicated. The protein levels of total MET proteins were detected by Western blotting with respective antibodies as described in panel A. Relative levels of total MET proteins during each treatment were quantified as in panel B. Shown is a representative set of blots from three independent repeats. (**E**) Serum-starved U373-MG cells were pulse-treated (7 minutes treatment) with either HGF (50 ng/ml), the biotin-conjugated anti-MET antibodies (2 μg/ml) or PBS (as control) as indicated. The cells were washed and then incubated in the starvation media to recover for the indicated times before harvesting. The protein levels of total MET protein were detected by Western blotting with anti-MET antibodies. Actin is used as the loading control. Relative levels of total MET proteins during each treatment at various time points were quantified as in panel B. Shown is a representative set of blots from three independent repeats. (**F**) Serum-starved U373-MG cells were pulse-treated for 7 minutes with either HGF (50 ng/ml), the biotin-conjugated anti-MET antibodies (2 μg/ml), or left untreated (control). The cells were washed extensively with warm PBS to remove the unbound HGF or the antibodies, and then incubated in the starvation media for recovery for the indicated times. Cells were then fixed and immunostained with anti-MET antibodies. Arrowheads indicate the presence of MET protein on the plasma membrane. Scale bars, 20um.

To determine the response of the MET protein to the ligand-dependent and ligand-independent activation stimuli, we measured the time course of a continuous treatment of serum-starved cells with HGF or the biotin-conjugated anti-MET antibodies (Fig. 6). We found that HGF stimulated rapid phosphorylation of the MET protein, reaching the peak level around 15 minutes after its addition to the serum-starved cells (Fig. 6A). Continued treatment of HGF for various time (30, 60, and 120 minutes) mostly maintained the total MET protein levels, with a partial reduction of the activated and phosphorylated form of MET at later time points. As shown by quantification in Fig. 6B,C, HGF treatment for 120 minutes did not significantly affect the total MET protein levels, although there is a gradual reduction of the phosphorylated MET protein levels. Furthermore, a continued exposure to HGF up to 24 hours caused minor changes in the levels of total MET proteins (Fig. 6D). Thus, prolonged treatment of HGF does not significantly affect the levels of total MET proteins (Fig. 6A,C,D).

In sharp contrast, however, the biotin-conjugated anti-MET antibodies induced MET protein phosphorylation with a similar kinetics as that of HGF, although slightly less potent (Fig. 6A,B). Continued exposure to the biotin-conjugated anti-MET antibodies led to a steady and faster decline of the phosphorylated form of MET protein and a decrease in the levels of the total MET protein starting at 30 minutes. The total MET protein, as well as the phosphorylated MET, completely disappeared by 120 minutes, in the antibody-treated cells (Fig. 6A–C). No significant effects on the levels of total MET protein were observed in cells treated with the biotin-conjugated control IgG (Fig. 6A). Similar effects were observed when cells were continuously treated with HGF or anti-MET antibodies for 4–24 hours (Fig. 6D).

We also examined the effects of pulse or transient stimulation with HGF or with anti-MET antibodies on the levels of total MET proteins, In these experiments, serum-starved cells were stimulated with HGF or anti-MET antibodies for 7 minutes (pulse), washed and then incubated in the fresh serum-starvation medium for various time (chase) (Fig. 6E). In cells that had been exposed to a 7-minute pulse of anti-MET antibodies, there was a rapid decline of the total MET protein levels (Fig. 6E). By 60 minutes of the chase, the level of total MET protein were reduced by 67%, and stayed at about this level through 240 minutes of the chase (Fig. 6E). The remaining 33% of the MET proteins, which were resistant to the anti-MET antibodies induced degradation, likely represented the intracellular pool of the MET proteins at the time before the exposure to antibodies (0 minute). In contrast, there was little reduction of total MET protein levels during the 240 minutes chase after a pulse-treatment with HGF (Fig. 6E), suggesting that the HGF-bound receptors can mostly recycle back to the cell surface. Using immunostaining, we found that in the cells that were pulse-stimulated with HGF, the MET protein was detected on the cell surface by 30 minutes of the chase, but in the cells that were pulse-stimulated with anti-MET antibodies, no cell surface MET protein was detected even after 120 minutes of the chase (Fig. 6F). Together, these observations suggest that while the MET RTKs activated by HGF can reappear on the cell surface, likely mediated through a recycling process, but the MET RTKs bound by the anti-MET antibodies were targeted to degradation.

### The biotin-conjugated anti-MET antibodies induce a proteome-sensitive and lysosome-dependent proteolysis of MET protein

**Figure 7:**
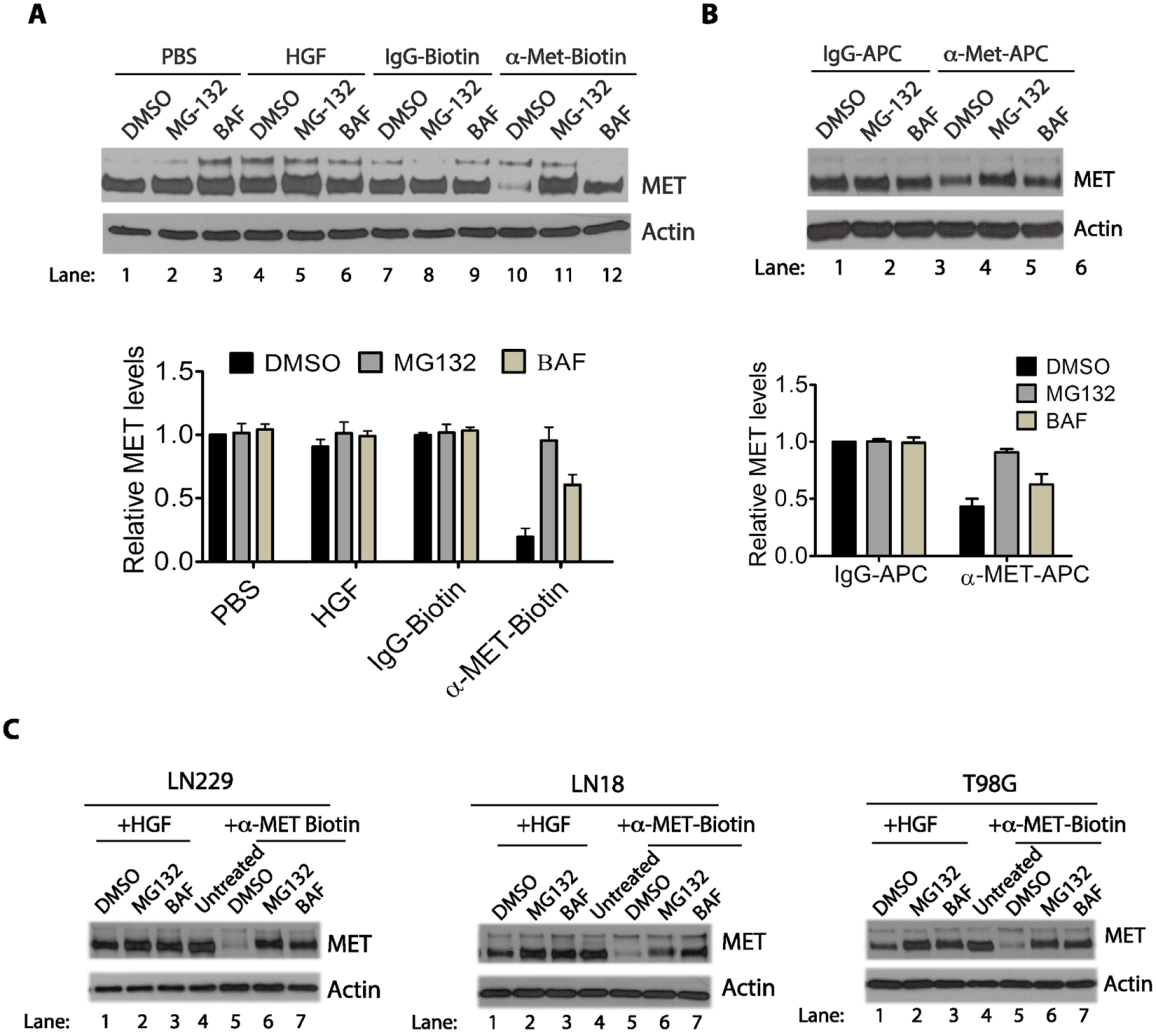
Biotin-conjugated anti-MET antibodies promote degradation of the MET protein in a proteasome-sensitive manner. (**A**) Serum-starved U373-MG cells were treated either with HGF (50 ng/ml) (lanes 4–6) or the biotin-conjugated anti-MET antibodies (2 μg/ml) (lanes 10–12), or with the corresponding controls PBS (lanes 1–3) or the control IgG (2 μg/ml) (lanes 7–9), for 7 minutes as indicated. To each set of the cells, either DMSO (control), MG-132 (10 μg/ml), or bafilomycin A1 (BAF, 100 nM) were then added and incubated for further 2 hours. The cells were lysed in SDS buffer and the protein levels of total MET protein were detected by Western blotting with anti-MET antibodies. Actin is used as the loading control. Relative levels of total MET proteins during each treatment were quantified normalized to the actin load control, and then calculated using the MET protein levels in the PBS/DMSO control sample (lane 1) as the reference point of 1.0. Data of quantitation is obtained from three independent repeats, and a set of blots from one of these experiments is shown. (**B**) Serum-starved U373-MG cells were treated either with APC-conjugated anti-MET antibodies (1 μg/ml, lanes 4–6) or control IgG (1 μg/ml, lanes 1–3) for 7 minutes as indicated. Cells were then treated with DMSO, MG-132 or BAF, as in panel A, and processed similarly as in panel A. Relative levels of total MET proteins during each treatment were quantified, normalized to the actin load control, and then calculated using the MET protein levels in the IgG-APC/DMSO control sample (lane 1) as the reference point of 1.0. Data of quantitation is obtained from three independent repeats, and a set of blots from one of these experiments is shown. (**C**) Serum-starved LN229, LN18 and T98G cells were treated either with biotin-conjugated anti-MET antibodies (2 μg/ml) (lanes 5–7) or HGF (50 ng/ml) (lanes 1–3) or left untreated (lane 4) for 7 minutes as indicated. Cells were then treated with DMSO, MG-132 or BAF, as in panel A. The cells were lysed in SDS buffer and the protein levels of total MET protein were detected by Western blotting with anti-MET antibodies. Actin is used as the loading control.

In normal cells, binding of HGF to MET is known to trigger mono-ubiquitination of the receptor proteins, with the mono-ubiquitination modification serving as a sorting signal to sort the marked receptors to multivesicular bodies (MVBs)^29–31^. MVBs can further mature into late endosomes, which then fuse with lysosomes, leading to degradation of the mono-ubiquitinated receptors in the lysosomes^32^. Interestingly, sorting of MET RTK in MVBs is also sensitive to proteasome inhibitors, although the exact mechanism remains unclear^29–31^. To examine the cause of MET protein disappearance in serum-starved cells treated with the biotin-conjugated anti-MET antibodies, we examined if the MET protein degradation could be prevented by MG-132, an inhibitor of the 26S proteosome that targets poly-ubiquitinated proteins for degradation^33^, or by bafilomycin A1 (BAF)^34^, a specific inhibitor of vacuolar-type H(+)-ATPase that inhibits the acidification of lysosomes and consequently blocks protein degradation in lysosomes. Serum-starved cells were treated with biotin-conjugated anti-MET antibodies or treated with HGF as control. After 7 minutes, BAF or MG-132 were added to the medium and incubated for further 2 hours (Fig. 7A). We found that the presence of MG-132 completely restored the MET protein levels in the cells treated with the biotin-conjugated anti-MET antibodies. In addition, BAF also partially stabilized the MET protein level in the cells treated with the biotin-conjugated anti-MET antibodies (Fig. 7A). As control, in the cells treated with HGF, a slight reduction (~5%) of MET protein levels following HGF treatment was observed, and such reduction was also prevented by MG-132 or BAF (Fig. 7A). Similar to the effects observed in cells treated with the biotin-conjugated goat anti-MET antibodies, MG-132 and BAF each rescued, or partially rescued, respectively, the MET levels in cells treated with the APC-conjugated mouse anti-MET antibodies (Fig. 7B). Similar effects of MG132 and BAF on preventing MET protein degradation induced by anti-MET antibodies were observed in several other GBM cells lines, including LN229, LN18 and TG98 (Fig. 7C). The anti-MET antibodies thus induced a proteasome-sensitive, and lysosome dependent, degradation of MET proteins that has been previously described for the HGF-induced degradation of MET proteins in other cells^29–31^, however, in U373-MG cells, HGF was completely ineffective in triggering down-regulation of MET proteins.

### The biotin-conjugated anti-MET antibodies inhibit cell migration

**Figure 8:**
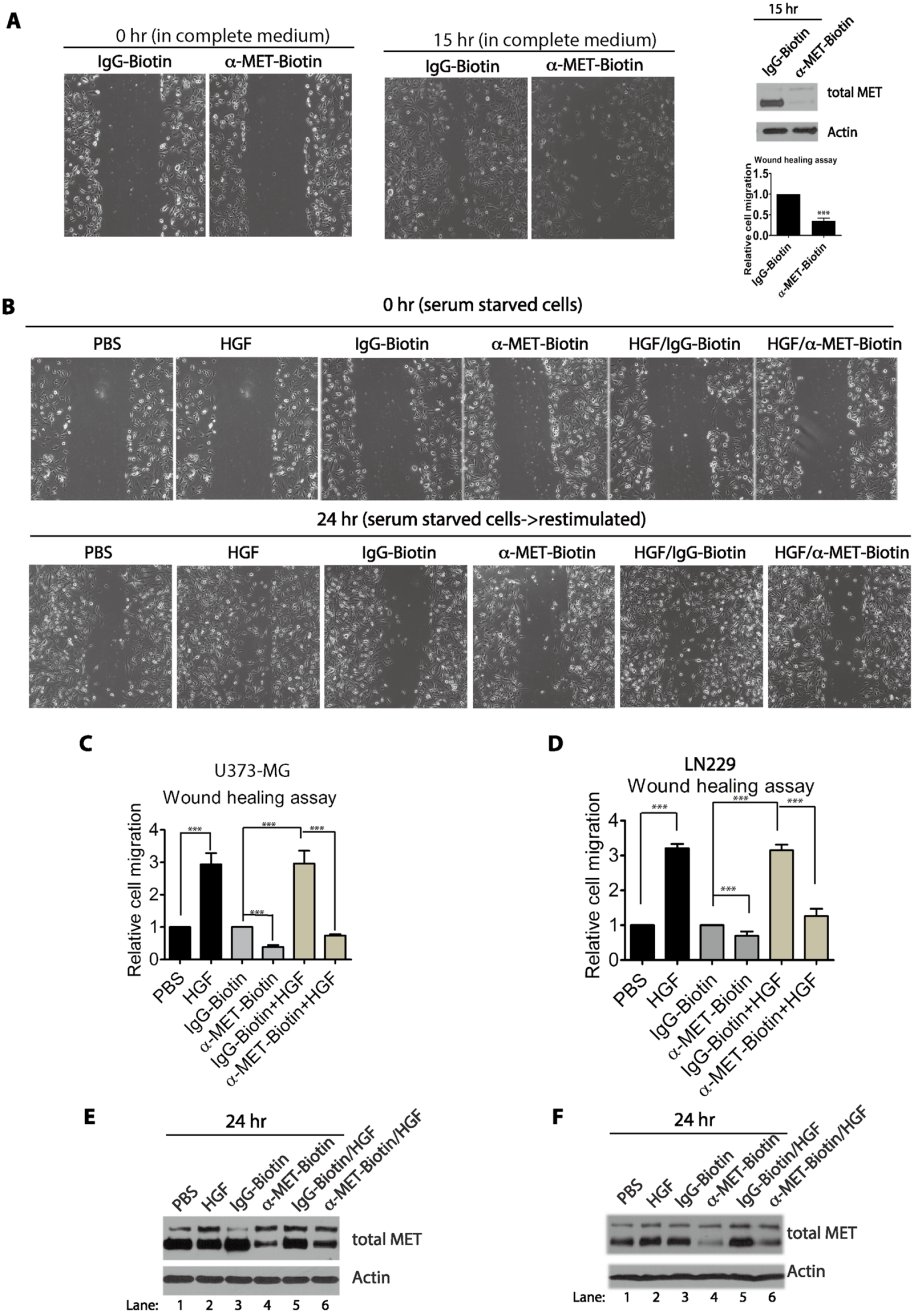
Biotin-conjugated anti-MET antibodies inhibited cell migration. (**A**) U373-MG cells were seeded on the plate and cultured in complete medium for 16 hours. A sterile 20 μl tip was used to scrape the cells to make a wound. The cells were washed with warm PBS to remove floating cells and replaced with fresh complete medium. The biotin-conjugated anti-MET antibodies or biotin-conjugated control IgG (each at 2 μg/ml) were added to the cells for additional 15 hours. The live cells were photographed (**A**). The cells migrated into the wound area at the 15 hour time point were quantified from 6 independent fields and integrated, and the means (with S.D.) from a duplicate set of samples were calculated. Statistical analysis was conducted (compared to IgG control) (statistical significance ***p < 0.001). The cells were then lysed in SDS buffer and the total levels of MET protein and actin (loading control) were examined by Western blotting. (**B,C,E**). U373-MG cells were seeded on the plate for 16 hours and then serum-starved for additional 9 hours. A scrape wound was made on each plate as in panel A (time point 0 hour). To each plate, HGF (50 ng/ml) or PBS (control) was added; alternatively, biotin-conjugated control antibodies (IgG-Biotin) or anti-MET antibodies (α-MET-Biotin) (each at 2 μg/ml), either alone or together with HGF, were added to the media; and cells were further incubated for 24 hours. The live cells were photographed at the time point 24 hours. The cells migrated into the wound area at the 24 hour time point were quantified as in panel A, and normalized to that when cells were treated with PBS and IgG control, and the means (with S.D.) from a duplicate set of samples were calculated and shown in panel C. Statistical analysis was conducted for each indicated pair (statistical significance ***p < 0.001). The U373-MG cells were lysed and the total levels of MET protein and actin (loading control) were examined by Western blotting (panel E). (**D,F**) The LN229 cells were treated in the same way and quantified as in panel B and C. Statistical analysis was conducted (compared to PBS and IgG control) (statistical significance ***p < 0.001). The cells were then lysed in SDS buffer and the total levels of MET protein and actin (loading control) were examined by Western blotting (panel F).

Because the anti-MET antibodies tested above trigger an efficient MET protein degradation, we wondered if exposure of cells to these antibodies can efficiently suppress the HGF-mediated biological events. Since the MET receptor plays a key role in cell migration and invasive growth of various cancer cells, we examined the potential effects of anti-MET antibodies on cell migration. In actively growing U373-MG cells, treatment of cells with the biotin-conjugated anti-MET antibodies for 15 hours potently suppressed the cell migration in a wound-healing assay, as compared to that of the biotin-conjugated control IgG (Fig. 8A). Western blot analysis confirmed that the MET protein levels are effectively down-regulated during the antibody treatment process (Fig. 8A). We also examined the effects of HGF and anti-MET antibodies in the serum-starved cells. While exposure to HGF for 24 hours significantly stimulated cell migration, similar exposure to the biotin-conjugated anti-MET antibodies could not stimulate cell migration. Rather, the antibodies in fact reduced the basal level of cell migration as compared to that of the biotin-conjugated IgG control (Fig. 8B,C). Notably, addition of both biotin-conjugated anti-MET antibodies and HGF to the serum-starved cells for 24 hours significantly blocked the stimulation activity of HGF on cell migration, while no inhibitory effects were observed with the biotin-conjugated control IgG (Fig. 8B,C). Under these conditions, the biotin-conjugated anti-MET antibodies markedly reduced the levels of total MET protein, even in the presence of HGF (Fig. 8E). Similar inhibitory effects of the biotin-conjugated anti-MET antibodies towards cell migration were also observed in other GBM cell lines, LN229, LN18 and T98G (Figs 8D,F; S5). Our studies thus indicate that the biotin-conjugated anti-MET antibodies can be used to target MET protein for degradation and block the biological activities of MET on cell migration in various GBM cells.

### The biotin-conjugated or APC-conjugated anti-MET antibodies inhibit cancer cell invasion

**Figure 9:**
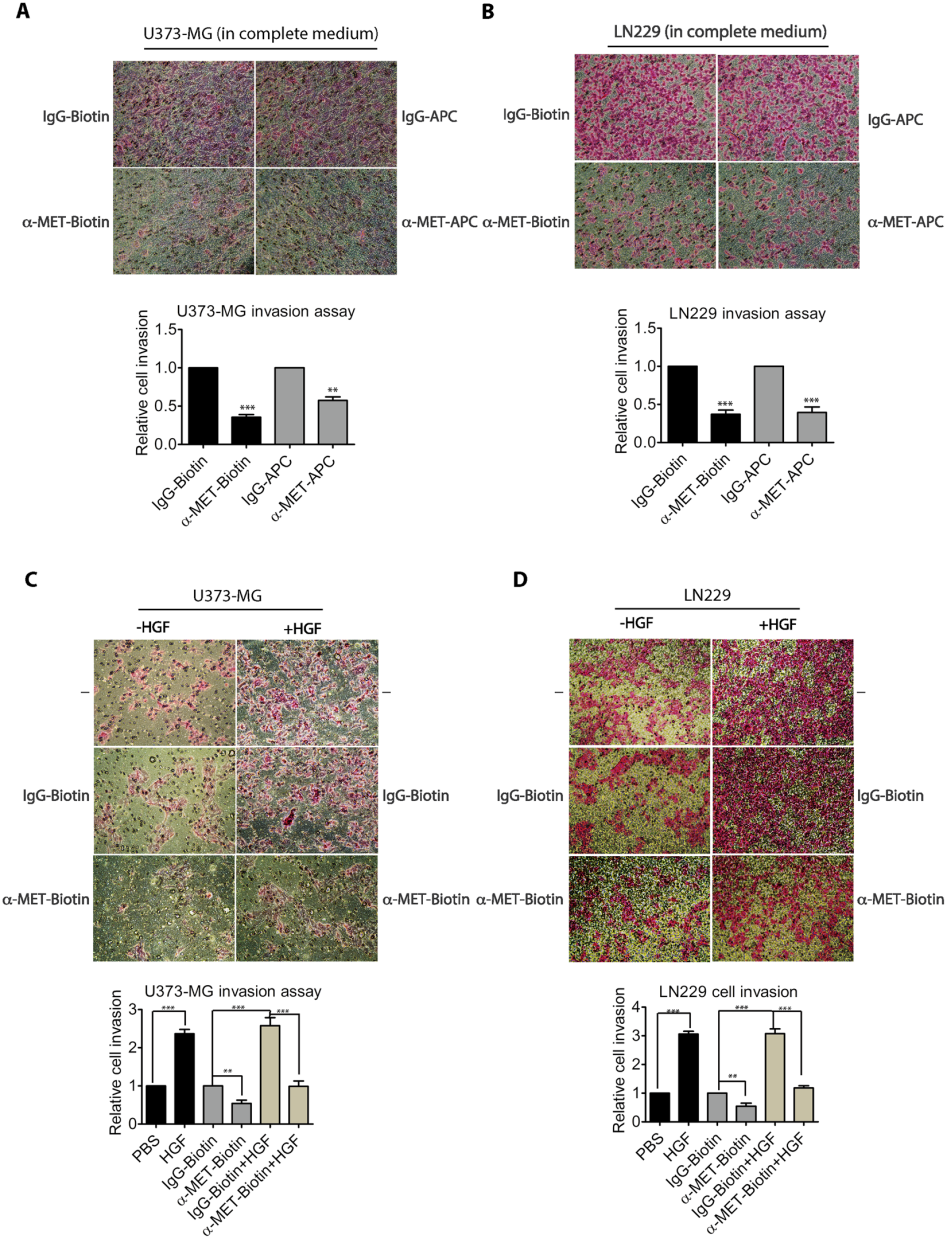
Biotin-conjugated anti-MET antibodies inhibited cell invasion. (**A**) An equal number of U373-MG cells were seeded to the top chamber of the Boyden chamber precoated with Matrigel. Cells were incubated in the complete medium and the bottom chamber also contained complete medium supplemented with biotin-conjugated control IgG or anti-MET antibodies (each at 2 μg/ml), or with APC-conjugated control IgG or anti-MET antibodies (each at 1 μg/ml). After 24 hours, cells that migrated to the bottom chamber (invaded through Matrigel) were stained, imaged by an EVOS light microscope, and quantified using the ImageJ software. Assays were performed in triplicates, and the means (with S.D.) are shown at the bottom. Statistical analysis was conducted (compared with the corresponding control in each subgroup) (***p < 0.001, **p < 0.01). (**B**) LN229 cells were assayed, similar to that described in panel A, except that the cells were allowed to invade for 32 hours. (**C**) U373-MG cells were starved for 9 hours and the equal number of cells were then seeded to the top chamber of the Boyden chamber with serum-starvation medium, and the bottom chamber contained the serum-starvation medium supplemented with HGF (50 ng/ml), PBS, anti-MET antibodies or control IgG as in panel A, or combination of HGF and antibodies as indicated. Invasion assays were carried out for 24 hours. The assay plates were then processed as in panel A and similarly quantified. Assays were performed in triplicates and the means (with S.D.) are shown on bottom. Statistical analysis was conducted (compared with the corresponding control in each subgroup) (***p < 0.001, **p < 0.01). (**D**) LN229 cells were assayed, similarly to that described in panel C, except that the cells were allowed to invade for 32 hours. Assays were performed in triplicates, and the means (with S.D.) are shown on bottom. Statistical analysis was conducted (compared with the corresponding control in each group) (***p < 0.001, **p < 0.01).

Glioblastoma cells are known to be highly invasive. To determine whether anti-MET antibodies affect such invasiveness, we further examined the effects of these anti-MET antibodies on gliobastoma cell invasion, using Transwells pre-coated with Matrigel (Fig. 9). We first assayed the effects of the anti-MET antibodies on cancer cell invasion when glioblastoma cells were cultured in the complete media. We have observed that the cancer cell invasion of both U373-MG and LN229 cells were effectively suppressed by either the biotin-conjugated goat anti-MET antibodies or the APC-conjugated mouse anti-MET antibodies (Fig. 9A,B). Quantification of the tumor cell invasion index showed that these antibodies can effectively reduce the cancer cell invasion index by 40–60%, depending on the antibodies used. We also assayed the effects of these antibodies on HGF-induced cancer cell invasion. Again, with both cell lines, we have observed that the biotin-conjugated anti-MET antibodies can effectively block HGF-induced cancer cell invasion (Fig. 9C,D). Our data indicate that these anti-MET antibodies are biologically inhibitory to the MET signaling pathway for cancer cell invasion.

## Discussion

In this report, we describe single cell-based assays to analyze the activation and subsequent processing of the MET receptor in response to the binding of antibodies in glioblastoma cells. By using fluorescently tagged anti-Met antibodies, we found that binding of anti-MET antibodies to the MET receptor induced the transient formation of a highly polarized receptor cluster on the plasma membrane, in a manner similar to the events when the receptors were bound by their cognate ligand, HGF (Fig. 1). However, while the HGF-bound MET receptors were internalized and then recycled to cell surface, as revealed by the limited degradation of MET following the HGF treatment, the antibody-bound MET receptors were rapidly degraded. A short exposure (7 minutes) to the anti-MET antibodies was sufficient to induce the degradation of the activated MET protein on the cell surface during the subsequent chase period (30–60 minutes), while a longer exposure (2 hours) to the antibodies led to the near complete depletion of the entire cellular pool of MET proteins (Fig. 6). We also found that the antibody-MET complexes were degraded through a process that is highly sensitive to the proteasome inhibitor MG-132 (Fig. 7). It is also partially sensitive to a proton pump inhibitor bafilomycin (Fig. 7). It is possible that the binding of MET with antibodies altered the highly efficient recycling pathway for MET proteins in glioblastoma cells, leading to degradation of the receptor proteins. The exact mechanisms by which the antibody-bound MET proteins are preferentially degraded await further studies.

We have recently found that HGF binding caused the formation of the MET-AXL complex and co-clustering of these receptors on the plasma membrane^22^. In the current study, we have tested two commercially available anti-MET antibodies, a goat polyclonal antibody (biotin-conjugated) and a mouse monoclonal antibody (APC-conjugated), which are likely to recognize different epitopes in the MET extracellular domain. However, both antibodies can efficiently induce the rapid MET-AXL co-clustering and the phosphorylation and activation of both MET and AXL (Figs 1 and 5). It remains unclear whether the MET-AXL co-clustering and AXL phosphorylation induced by the anti-MET antibodies is mediated through the formation of the MET-MET homodimers which then lead to the formation of the MET-AXL heterodimers or whether these antibodies directly activate the MET protein in the MET-AXL heterodimer complex through binding to MET. Nevertheless, both biotin-conjugated and APC-conjugated antibodies caused an altered processing of the activated MET proteins, leading to the rapid degradation of the receptors, instead of recycling of these receptors when binding to HGF (Figs 6 and 7). These observations suggest that these two types of antibodies may induce a common structural or conformation change in MET to alter the receptor trafficking routes.

The MET RTK is often over-expressed in human cancers, and the activation of MET RTK is associated with aggressive cancer cell growth, metastasis and resistance towards a variety of anti-cancer drugs^3,4,6^. How to effectively target the over-expressed or abnormally activated MET proteins is an important question for developing novel anti-cancer therapeutics. Several anti-HGF antibodies are in the pre-clinical or clinical development, and these antibodies act by neutralizing the ligand for the receptor or preventing the ligand binding to the receptor^15,16^. Alternatively, several anti-MET monoclonal antibodies have been developed to block the interaction of MET with HGF. Since one major concern is that bivalent antibodies may activate MET, substantial efforts were aimed to identify the non-agonist antibodies such as the monovalent antibodies. One example is the one-armed monovalent 5D5 antibody^17–19^, which binds to the monomeric form of MET. Although this antibody blocks the binding of HGF to the receptor, a phase III clinical trial for this antibody has produced disappointing results due to its lack of clinical efficacy^35^. A bivalent antibody that does not activate the RTK activity of MET, SAIT301, was developed and reported to cause a CBL-independent downregulation of MET after an incubation of cells with antibodies for 24 hours^21^. Another bivalent monoclonal antibody, LY2875358, was recently shown to possess a neutralizing activity against HGF through blocking the interaction between HGF and MET^20^. It was also observed that LY2875358 caused a slow and lysosome-dependent degradation of MET, with 50% reduction of the MET protein level occurred after 24 hours of incubation^20^. Contrary to the concern that bivalent antibodies may activate the targeted RTKs, in this report, we found that glioblastoma cells have an unusual high activity of HGF-induced MET receptor recycling with very few MET molecules get downregulated after HGF stimulation. Treatment of these cancer cells with the bivalent anti-MET antibodies induces only a transient activation of MET RTK activities. What follows is an altered intracellular processing of the MET protein through a novel ubiquitin-dependent degradation process involving lysosomes that causes the rapid depletion of the near entire pool of MET proteins. Consequently, these anti-MET bivalent antibodies are biologically inhibitory for cancer cell migration and invasion. Our studies suggest that development of bivalent anti-MET immuno-reagents may hold a promise to target and remove the MET proteins from glioblastoma cells or other cancer cells that possess high HGF-induced recycling activity of MET.

## Methods

### Cells, antibodies, and reagents

Human glioblastoma cell lines U373-MG, T98G, LN229 and LN18 were purchased from American Type Culture Collection (ATCC). Recombinant human HGF was from Invitrogen/Thermo Fisher. Antibodies against MET, including goat anti-MET antibody (biotin-conjugates, #BAF358), and mouse monoclonal anti-MET antibody (APC-conjugate, clone #95106, #FAB3528P), and control goat IgG (biotin-conjugates) and control mouse IgG (APC-conjugate), antibodies against MET (goat, #AF276), phosphorylated AXL (p-779-AXL, rabbit, #AF2228) were from R&D Systems. Rabbit antibodies against AXL, MET, Y1234/Y1235-phosphorylated MET, phosphorylated AKT (at S473, recognizing all AKT isoforms, Cat. #9272), and anti-MET rabbit monoclonal antibody (clone D1C2, Alexa Fluor 488 conjugate, Cat. #8494) were obtained from Cell Signaling Technology. Chemicals, MG-132 and bafilomycin, were from Sigma-Aldrich. Secondary antibodies, including the Alexa Fluor 488-conjugated goat anti-mouse IgG, Alexa Fluor 647-conjugated goat anti-mouse IgG and Alexa Fluor 647-conjugated goat anti-rabbit IgG antibodies were from Jackson Immunologicals. Brilliant Violet 421 or 605-conjugated streptavidin was from BioLegend. The CellTiter 96 AQueous One Solution Kit was from Promega and the Alexa Fluor 488 Annexin V/Dead Cell Apoptosis Kit was from Invitrogen/Thermo Fisher. For tyrosine kinase inhibitors of MET, crizotinib was obtained from Cell Signaling Technology; JNJ-38877605 and SGX-523 were obtained from Selleck Chemicals. Boyden chambers with 8 μm pores were from Costar (Cambridge, MA), and the growth factor-reduced Matrigel was from BD Biosciences. Differential Quik Stain Kit was obtained from Electron Microscopy Sciences.

### Cell lysis and Western blot analysis

Cells were lysed in lysis buffer containing 50 mMTris (pH 7.4), 150 mM NaCl, 1% Triton-X-100, 0.1% SDS, supplemented with 5 mM NaF, 1 mM Na3VO4, and 1X complete protease inhibitor (Roche, Indianapolis, IN). Western blotting was conducted as previously described^36^.

### Immunostaining and fluorescence microscopy

U373-MG cells were serum-starved for 3 hours, and the starved cells were then stimulated with or without HGF (50 ng/ml), APC-conjugated mouse monoclonal anti-MET antibody (1 μg/ml) and biotin-conjugated anti-MET antibody (2 μg/ml) for 7 minutes before fixation. Immunofluorescence experiments were conducted as described^23^. Briefly, cells were fixed and permeabilized with Fixation and Permeabilization Solution (BD Biosciences), blocked with goat serum, incubated with primary antibodies and then secondary antibodies in phosphate-buffered saline (PBS) containing 0.05% Tween 20 and 7% goat serum. Images were acquired using a Nikon A1R confocal laser scanning microscopy system equipped with the NIS-Elements Advance Research software with 20x objective (NA 0.8). For double or triple immunostaining, fluorescence were obtained after excitation with laser light of 405 nm, 488 nm and 640 nm to capture images for Brilliant Violet 421 (laser 405), Alexa Fluor 488 and Alexa Fluor 647, respectively, and these fluorophores have minimal fluorescence bleed-throughs. Exposure settings were kept constant for all conditions within each set of experiments. The captured images were further analyzed and quantified by using the NIS Elements Viewer software.

### Wound healing assay

U373-MG cells were seeded on the plate and cultured for 16 hours. A sterile 20 μl tip was used to scrape the cells to make a wound. The cells were washed twice with warm PBS to remove floating cells. The biotin-conjugated anti-MET antibody or Biotin-conjugated IgG (each at 2 μg/ml) were added to the cells for additional 15 hours or longer, depending on the specific cell lines. For cells to be treated with HGF or anti-MET-biotin antibody stimulated assay, cells were starved for 9 hours, then stimulated for 24 hours with HGF (50 ng/ml) or anti-MET antibodies (2 μg/ml) or the corresponding controls. Cells were photographed using the EVOS XL Core microscope system with 20x objective and the cells migrated into the wound area were quantified using the Image J software^37^. For each condition, cells were counted from 6 independent fields and integrated. All experiments were done in duplicates.

### Cell invasion assay

Cell invasion assays were performed using Boyden chambers with 8 μm pores, coated with the growth factor-reduced Matrigel diluted in Dulbecco’s modified Eagle’s medium. Cells were detached by trypsin and washed once with starvation medium (Dulbecco’s modified Eagle’s medium, 0.5% fetal bovine serum). Twenty thousand cells were seeded in the upper chamber for each condition in starvation medium, and cells were allowed to invade for 24 hours toward the lower chamber containing starvation medium supplemented with HGF (50 ng/ml) or PBS or IgG or anti-MET antibodies (2 μg/ml for biotin-conjugated antibodies, 1 μg/ml for APC-conjugated antibodies), or in combinations of HGF + anti-MET antibodies or corresponding controls. For cell invasion assay in the complete medium, cells were seed on the upper chamber, and cells were allowed to invade for 24 hours toward the lower chamber containing complete medium supplemented with IgG or anti-MET antibodies. Cells were then fixed with 4% paraformaldehyde. Cells in the upper chambers were mechanically removed using cotton swabs. Cells invaded into the lower chambers were then stained with the Differential Quik Stain Kit (modified Giemsa stain, obtained from Electron Microscopy Sciences). Cells were imaged using an EVOS XL Core system with 20x objective and quantified using the ImageJ software. For each condition, cells were counted from 6 independent fields and integrated. All experiments were done in duplicates.

### Statistics and bioinformatics

Quantitative data are expressed as the mean ± S.D. (S.D., standard deviation), normalized to the control condition, and plots were prepared using the GraphPad Prism 5 software. Statistically significant differences between means were determined using a two-tailed equal-variance Student’s t-test^38^. Different data sets were considered to be statistically significant when the p-value was 0.001 (***), 0.01 (**) or <0.05 (*).

## Supplementary information

Supplementary Information

## References

[1] Cooper CS (1984). Molecular cloning of a new transforming gene from a chemically transformed human cell line. Nature.

[2] Rodrigues GA, Park M (1993). Dimerization mediated through a leucine zipper activates the oncogenic potential of the met receptor tyrosine kinase. Molecular and cellular biology.

[3] Birchmeier C, Birchmeier W, Gherardi E, Vande Woude GF (2003). Met, metastasis, motility and more. Nature reviews. Molecular cell biology.

[4] Trusolino L, Bertotti A, Comoglio PM (2010). MET signalling: principles and functions in development, organ regeneration and cancer. Nature reviews. Molecular cell biology.

[5] Comoglio PM, Giordano S, Trusolino L (2008). Drug development of MET inhibitors: targeting oncogene addiction and expedience. Nature reviews. Drug discovery.

[6] Maroun CR, Rowlands T (2014). The Met receptor tyrosine kinase: a key player in oncogenesis and drug resistance. Pharmacology & therapeutics.

[7] Koochekpour S (1997). Met and hepatocyte growth factor/scatter factor expression in human gliomas. Cancer research.

[8] Maher EA (2001). Malignant glioma: genetics and biology of a grave matter. Genes & development.

[9] Barrow-McGee R, Kermorgant S (2014). Met endosomal signalling: in the right place, at the right time. The international journal of biochemistry & cell biology.

[10] Joffre C (2011). A direct role for Met endocytosis in tumorigenesis. Nature cell biology.

[11] Rose SD, Aghi MK (2010). Mechanisms of evasion to antiangiogenic therapy in glioblastoma. Clinical neurosurgery.

[12] Jahangiri A (2013). Gene expression profile identifies tyrosine kinase c-Met as a targetable mediator of antiangiogenic therapy resistance. Clinical cancer research: an official journal of the American Association for Cancer Research.

[13] Engelman JA (2007). MET amplification leads to gefitinib resistance in lung cancer by activating ERBB3 signaling. Science.

[14] Lin L, Bivona TG (2012). Mechanisms of Resistance to Epidermal Growth Factor Receptor Inhibitors and Novel Therapeutic Strategies to Overcome Resistance in NSCLC Patients. Chemotherapy research and practice.

[15] Eder JP, Vande Woude GF, Boerner SA, LoRusso PM (2009). Novel therapeutic inhibitors of the c-Met signaling pathway in cancer. Clinical cancer research: an official journal of the American Association for Cancer Research.

[16] Lee D (2015). Development of antibody-based c-Met inhibitors for targeted cancer therapy. ImmunoTargets and therapy.

[17] Martens T (2006). A novel one-armed anti-c-Met antibody inhibits glioblastoma growth *in vivo*. Clinical cancer research: an official journal of the American Association for Cancer Research.

[18] Jin H (2008). MetMAb, the one-armed 5D5 anti-c-Met antibody, inhibits orthotopic pancreatic tumor growth and improves survival. Cancer research.

[19] Merchant M (2013). Monovalent antibody design and mechanism of action of onartuzumab, a MET antagonist with anti-tumor activity as a therapeutic agent. Proceedings of the National Academy of Sciences of the United States of America.

[20] Liu L (2014). LY2875358, a neutralizing and internalizing anti-MET bivalent antibody, inhibits HGF-dependent and HGF-independent MET activation and tumor growth. Clinical cancer research: an official journal of the American Association for Cancer Research.

[21] Lee JM (2014). Cbl-independent degradation of Met: ways to avoid agonism of bivalent Met-targeting antibody. Oncogene.

[22] Li W (2018). HGF-induced formation of the MET-AXL-ELMO2-DOCK180 complex promotes RAC1 activation, receptor clustering, and cancer cell migration and invasion. The Journal of biological chemistry.

[23] Zhu L (2016). Acid sphingomyelinase is required for cell surface presentation of Met receptor tyrosine kinase in cancer cells. Journal of cell science.

[24] Harder T (2012). The T Cell Plasma Membrane Lipid Bilayer Stages TCR-proximal Signaling Events. Frontiers in immunology.

[25] Straussman R (2012). Tumour micro-environment elicits innate resistance to RAF inhibitors through HGF secretion. Nature.

[26] Zou HY (2007). An orally available small-molecule inhibitor of c-Met, PF-2341066, exhibits cytoreductive antitumor efficacy through antiproliferative and antiangiogenic mechanisms. Cancer research.

[27] Finisguerra V (2015). MET is required for the recruitment of anti-tumoural neutrophils. Nature.

[28] Buchanan SG (2009). SGX523 is an exquisitely selective, ATP-competitive inhibitor of the MET receptor tyrosine kinase with antitumor activity *in vivo*. Molecular cancer therapeutics.

[29] Jeffers M, Taylor GA, Weidner KM, Omura S, Vande Woude GF (1997). Degradation of the Met tyrosine kinase receptor by the ubiquitin-proteasome pathway. Molecular and cellular biology.

[30] Hammond DE, Urbe S, Vande Woude GF, Clague MJ (2001). Down-regulation of MET, the receptor for hepatocyte growth factor. Oncogene.

[31] Hammond DE (2003). Endosomal dynamics of Met determine signaling output. Molecular biology of the cell.

[32] Woodman PG, Futter CE (2008). Multivesicular bodies: co-ordinated progression to maturity. Current opinion in cell biology.

[33] Tsubuki S, Saito Y, Tomioka M, Ito H, Kawashima S (1996). Differential inhibition of calpain and proteasome activities by peptidyl aldehydes of di-leucine and tri-leucine. Journal of biochemistry.

[34] Yoshimori T, Yamamoto A, Moriyama Y, Futai M, Tashiro Y (1991). Bafilomycin A1, a specific inhibitor of vacuolar-type H(+)-ATPase, inhibits acidification and protein degradation in lysosomes of cultured cells. The Journal of biological chemistry.

[35] Spigel DR (2017). Results From the Phase III Randomized Trial of Onartuzumab Plus Erlotinib Versus Erlotinib in Previously Treated Stage IIIB or IV Non-Small-Cell Lung Cancer: METLung. Journal of clinical oncology: official journal of the American Society of Clinical Oncology.

[36] Zhang H, Kobayashi R, Galaktionov K, Beach D (1995). p19Skp1 and p45Skp2 are essential elements of the cyclin A-CDK2 S phase kinase. Cell.

[37] Schindelin J (2012). Fiji: an open-source platform for biological-image analysis. Nature methods.

[38] Fay, D. S. & Gerow, K. A biologist’s guide to statistical thinking and analysis. *WormBook: the online review of C. elegans biology*, 1–54, 10.1895/wormbook.1.159.1 (2013). 10.1895/wormbook.1.159.1 PMC3880567 23908055

