# Supplementary material for: Induction of MET Receptor Tyrosine Kinase Down-regulation through Antibody-mediated Receptor Clustering
Source: Sci Rep. 2019 Feb 13;9:1988. doi: 10.1038/s41598-018-36963-3 (PMC6374517; doi:10.1038/s41598-018-36963-3)
Supplement: Supplementary file 1 — Supplementary Information [file 41598_2018_36963_MOESM1_ESM.pdf]

Supplementary Information

Induction of MET Receptor Tyrosine Kinase Down-regulation through Antibody-mediated Receptor Clustering

Wenjing Li[1,2], Adam Dick[2], Fei Lu[1], Hui Zhang[2], and Hong Sun[2*]

From [1]School of Chemical Biology and Biotechnology, Peking University Shenzhen Graduate School, Shenzhen, Guangdong, China

[2]Department of Chemistry and Biochemistry, University of Nevada, Las Vegas, Las Vegas, NV 89154-4003, USA

[*]To whom correspondence should be addressed: Dr. Hong Sun, Department of Chemistry and Biochemistry, University of Nevada, 4505 S. Maryland Parkway, SEB 4138, Las Vegas, NV89154, Telephone: 702-774-1485, Fax: 702-895-4072, E-mail: hong.sun@unlv.edu

Suppl Figure 1

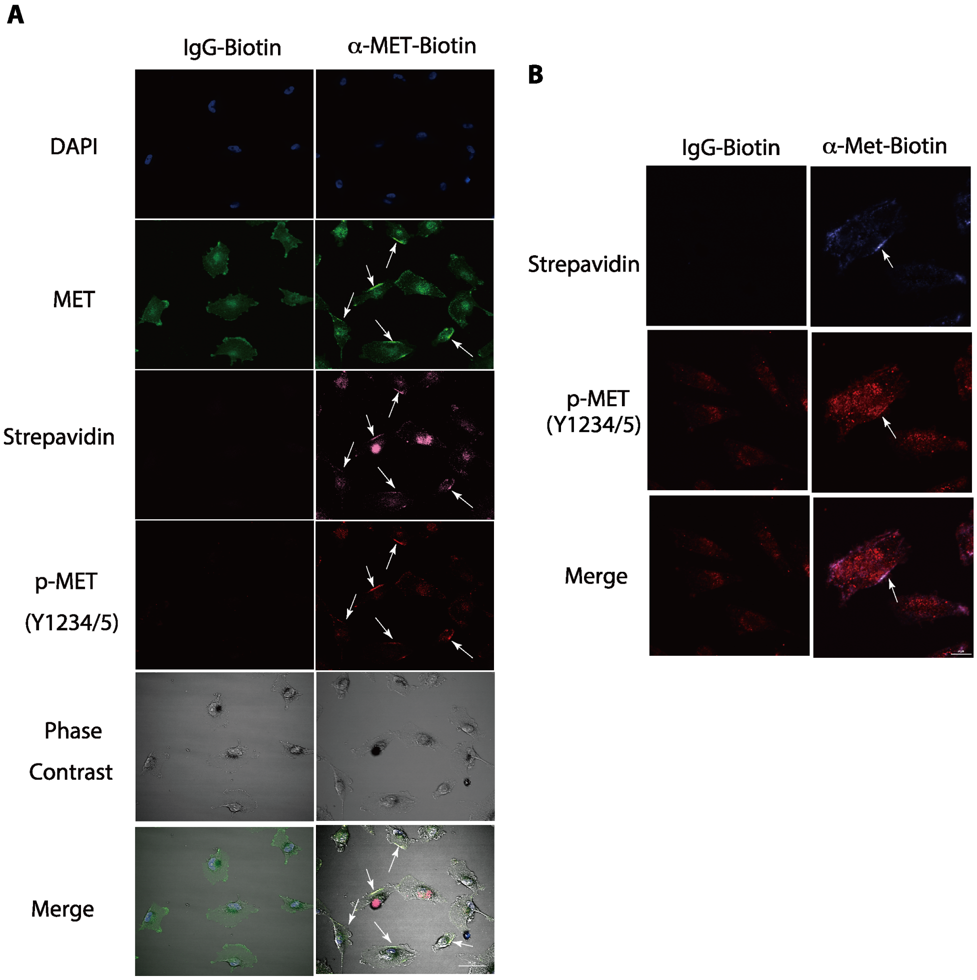

Suppl Figure 1. Anti-MET antibodies induce to the receptor clustering and patch formation on the plasma membrane through activation of MET receptor.

A. Live serum-starved U373-MG cells were treated with biotin-conjugated goat anti-MET antibodies or biotin-conjugated normal goat IgG antibodies (control), each at 2 μg/ml, for 7 minutes. Cells were then washed, fixed and immunostained with the Streptavidin-conjugated to Brilliant Violet 605 to detect the biotin-conjugated antibodies (pink), a rabbit anti-Y1234/1235-phosphorylated MET antibody for the activated MET protein (secondary antibody conjugated with Alexa Fluor 647, red), and an anti-MET antibody pre-conjugated to Alexa Fluor 488 for total MET protein (green). Cells were also counter-stained with DAPI to view nucleus. Phase contrast images were also taken to see cell contours. Scale bars, 50 μm.

B. Live serum-starved T98G cells were treated with biotin-conjugated goat anti-MET antibodies or biotin-conjugated normal goat IgG antibodies (control), each at 2 μg/ml, for 7 minutes. Cells were then washed, fixed and immunostained with the Streptavidin-conjugated to Brilliant Violet 421 to detect the biotin-conjugated antibodies (purple) and a rabbit anti-Y1234/1235-phosphorylated MET antibody for the activated MET protein (secondary antibody conjugated with Alexa Fluor 647, red). Scale bars, 20 μm.

Suppl Figure 2

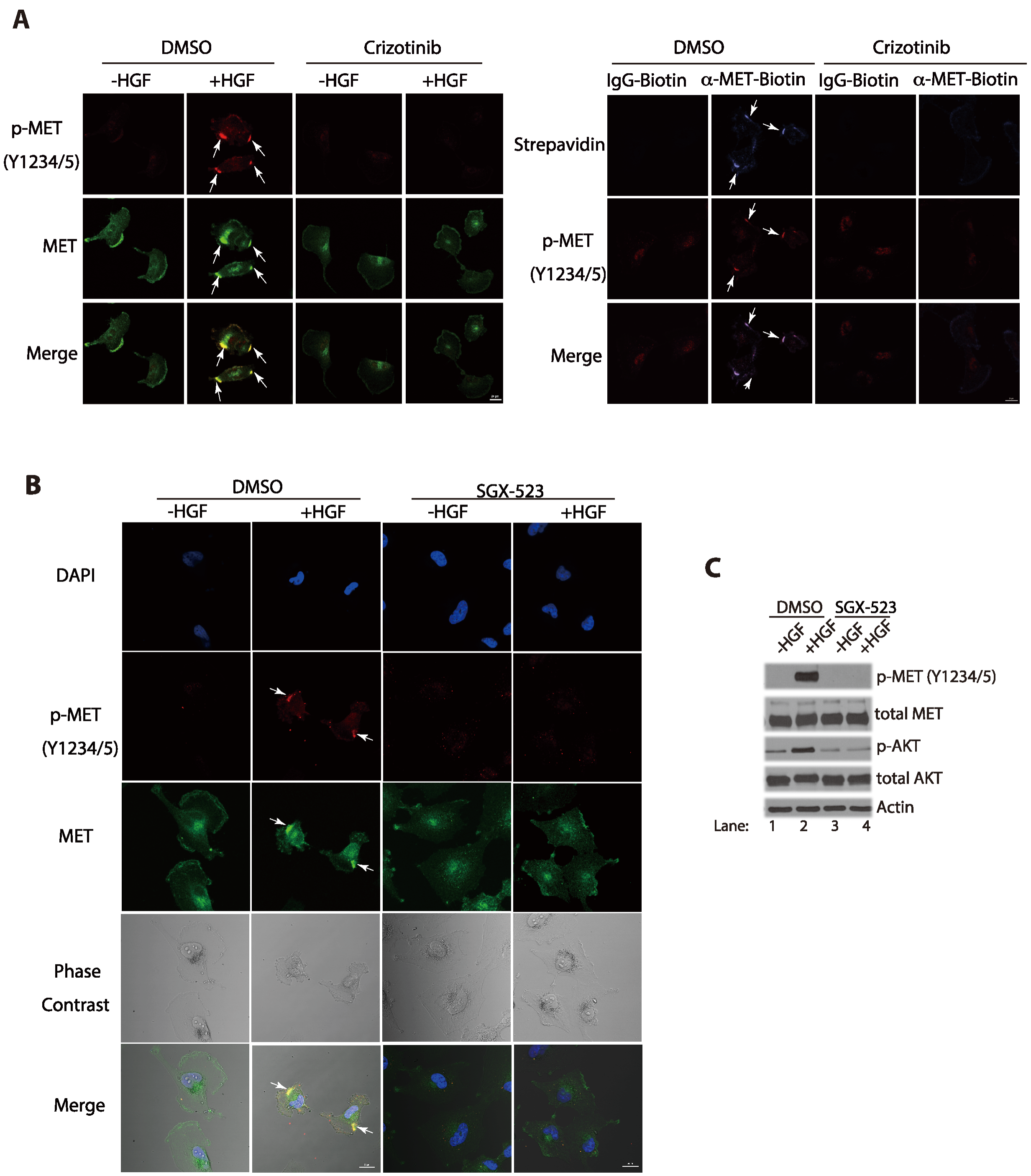

Suppl Figure 2. Both Crizotinib and SGX-523 inhibit the HGF-dependent or antibody-induced MET RTK activities.

A. (Left panels) Serum-starved U373-MG cells were treated with the MET kinase inhibitor, crizotinib (1 μM) or vehicle control dimethylsulfoxide (DMSO) for 3 hours and then stimulated with HGF (50 ng/ml) for 7 minutes. Cells were fixed and immunostained with a rabbit antibody against Y1234/Y1235-phosphorylated MET for the activated MET protein (red) and with an anti-MET antibody pre-conjugated with the Alexa-Fluor-

488 (green) to detect total MET. MET receptor clusters (patches) are indicated by arrows. (Right panels) Serum-starved U373-MG cells were treated with the MET inhibitor, crizotinib (1 μM), or dimethylsulfoxide (DMSO) vehicle control for 3 hours and the live cells were then treated with biotin-conjugated goat anti-MET antibodies or biotin-conjugated goat IgG antibodies (control), each at 2 μg/ml, for 7 minutes. The cells were then fixed and stained with Streptavidin-conjugated to Brilliant Violet 421 to detect the biotin-conjugated antibodies (purple) and the rabbit anti-Y1234/1235-phosphorylated MET antibodies (followed by secondary antibodies conjugated to the Alexa Fluor 647) for the activated MET proteins (red). MET receptor clusters (patches) are indicated by arrows. Scale bars, 20 μm.

B. Serum-starved U373-MG cells were treated with the MET kinase inhibitor, SGX-523 (1 μM) or dimethylsulfoxide (DMSO) vehicle control for 3 hours. The cells were stimulated with or without HGF (50 ng/ml) for 7 minutes and were subsequently fixed and stained with rabbit antibody against Y1234/Y1235-phosphorylated MET (red) for the activated MET proteins and with the Alexa-Fluor 488 pre-conjugated anti-MET antibody for total MET proteins (green). Nucleus was counter-stained with DAPI. Phase contrast pictures were also taken to view the cell contours. MET receptor clusters (patches) are indicated by arrows. Scale bars, 20 μm.

C. Serum-starved U373-MG cells were treated with SGX-523 (1 μM) or DMSO vehicle control as in B and then directly lysed in SDS buffer and proteins were detected by Western blotting with the indicated antibodies.

Suppl Figure 3

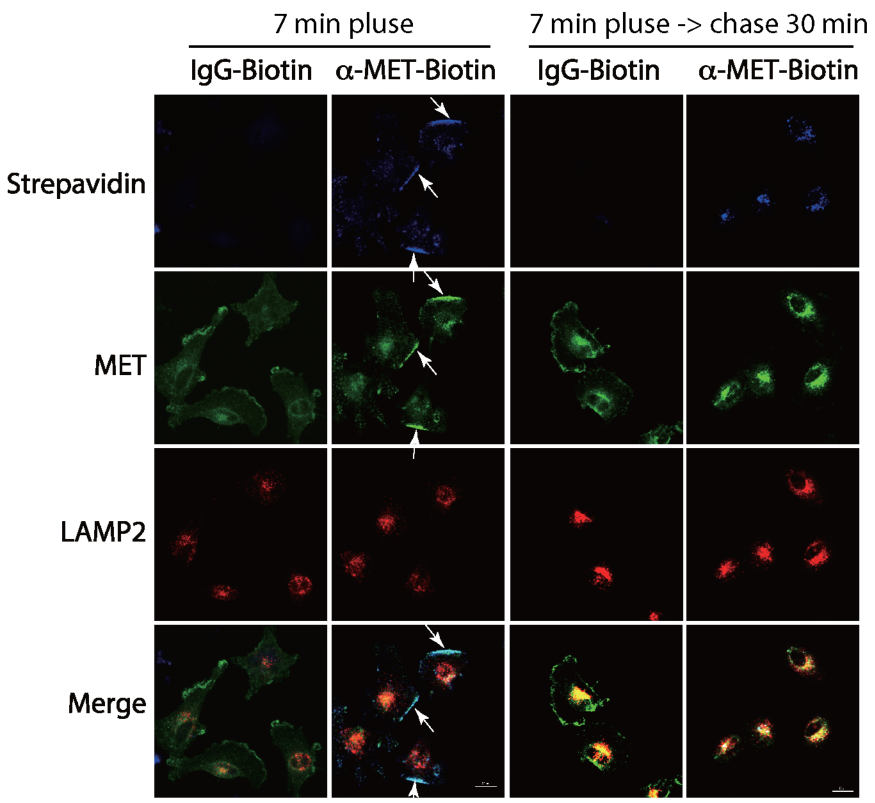

Suppl Figure 3. Anti-MET-biotin antibody causes accumulation of MET in lysosome.

The live serum-starved U373-MG cells were pulse treated with biotin-conjugated goat anti-MET antibodies or biotin-conjugated goat IgG antibodies (control), each at 2 μg/ml, for 7 minutes. The antibodies were washed out and chase for additional 30 minutes. The cells were then fixed and stained with Streptavidin labeled with Brilliant Violet 421 for biotin-conjugated antibodies (purple), the Alexa-Fluor 488 pre-conjugated anti-MET antibody for total MET protein (green), and an anti-LAMP2 antibody (pre-conjugated to Alexa-Fluor 647) to mark the lysosomes. MET receptor clusters (patches) are indicated by arrows. Scale bars, 20 μm.

Suppl Figure 4

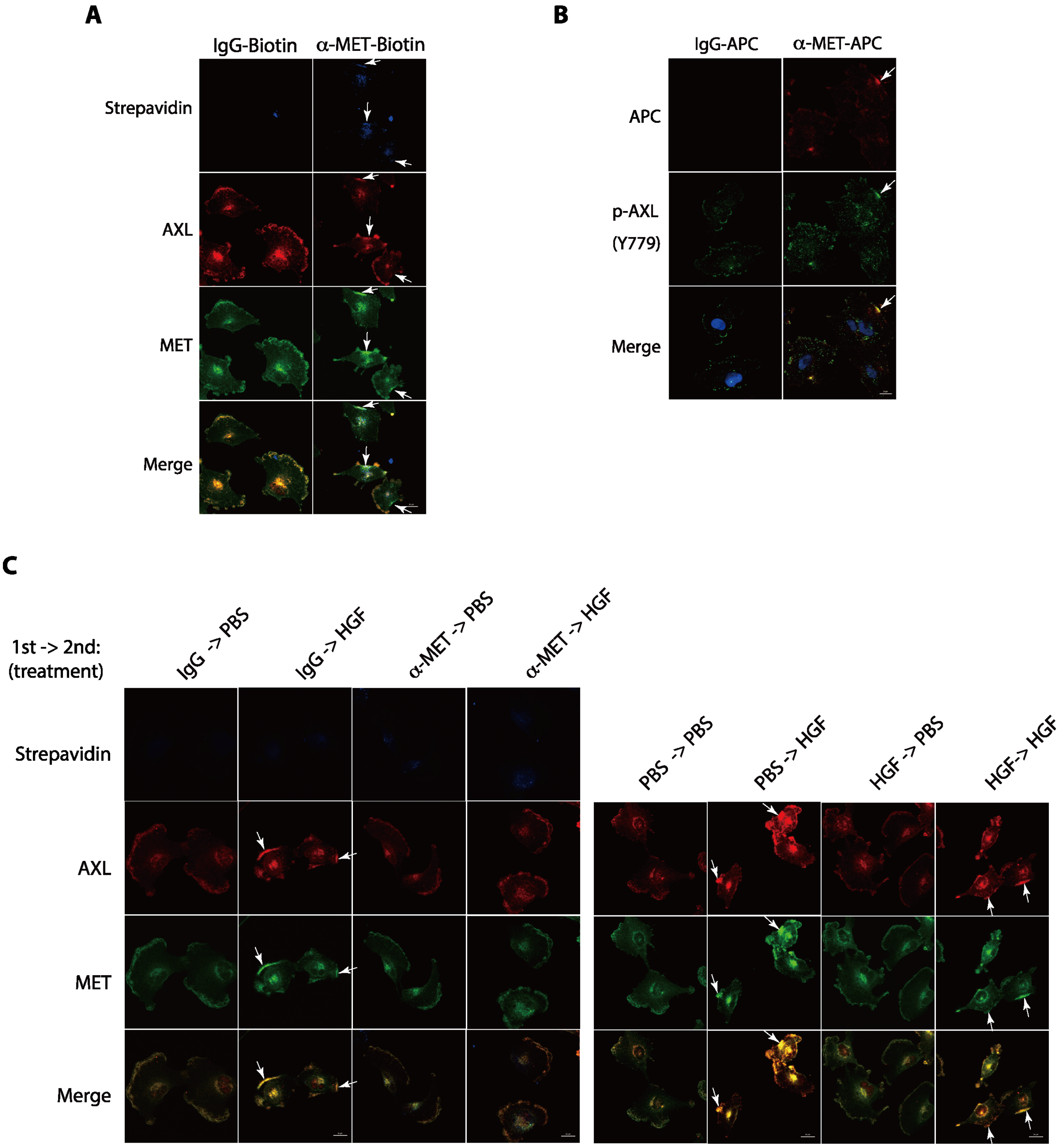

Suppl Figure 4. Effects of anti-MET-Biotin antibody and anti-MET-APC antibody on AXL activation. A. Live serum-starved U373-MG cells were treated with biotin-conjugated goat anti-MET antibodies or biotin-conjugated normal goat IgG antibodies (control), each at 2 μg/ml, for 7 minutes. Cells were then washed, fixed and immunostained with the Streptavidin-conjugated to Brilliant Violet 421 to detect the biotin-conjugated antibodies (purple), rabbit AXL antibody for total AXL protein (followed by a secondary antibody conjugated

with Alexa Fluor 647, red), and an anti-MET antibody pre-conjugated to Alexa Fluor 488 for total MET protein (green). MET receptor clusters (patches) are indicated by arrows. Scale bars, 20 μm.

B. Live U373-MG cells, which were serum starved for three hours, were incubated with the APC-conjugated mouse monoclonal anti-MET antibody (red) or APC-conjugated isotype control normal mouse IgG, each at 1 μg/ml also for 7 minutes. Cells were then washed, fixed and co-immunostained with rabbit anti-Y779-phosphorylated AXL antibody (followed by a secondary antibody conjugated with Alexa Fluor 488, green). The MET and AXL receptor clusters (patches) are indicated by arrows. Scale bars, 20 μm.

C. U373-MG cells were serum-starved for 3 hours. Cells were pulse-treated (1st treatment) with the biotin-conjugated anti-MET antibodies (2 μg/ml) or biotin-conjugated IgG (2 μg/ml) or PBS (phosphate buffered saline, control) or HGF (50 ng/ml) for 7 minutes, washed extensively with warm PBS buffer, and then incubated in the serum-starvation medium for 30 minutes to recover. They were then re-stimulated (2nd treatment) with HGF or PBS (as control) for another 7 minutes. Cells were fixed and immunostained with the anti-AXL or anti-MET antibodies. MET and AXL receptor clusters (patches) are indicated by arrows. Scale bars, 20 μm.

Suppl Figure 5

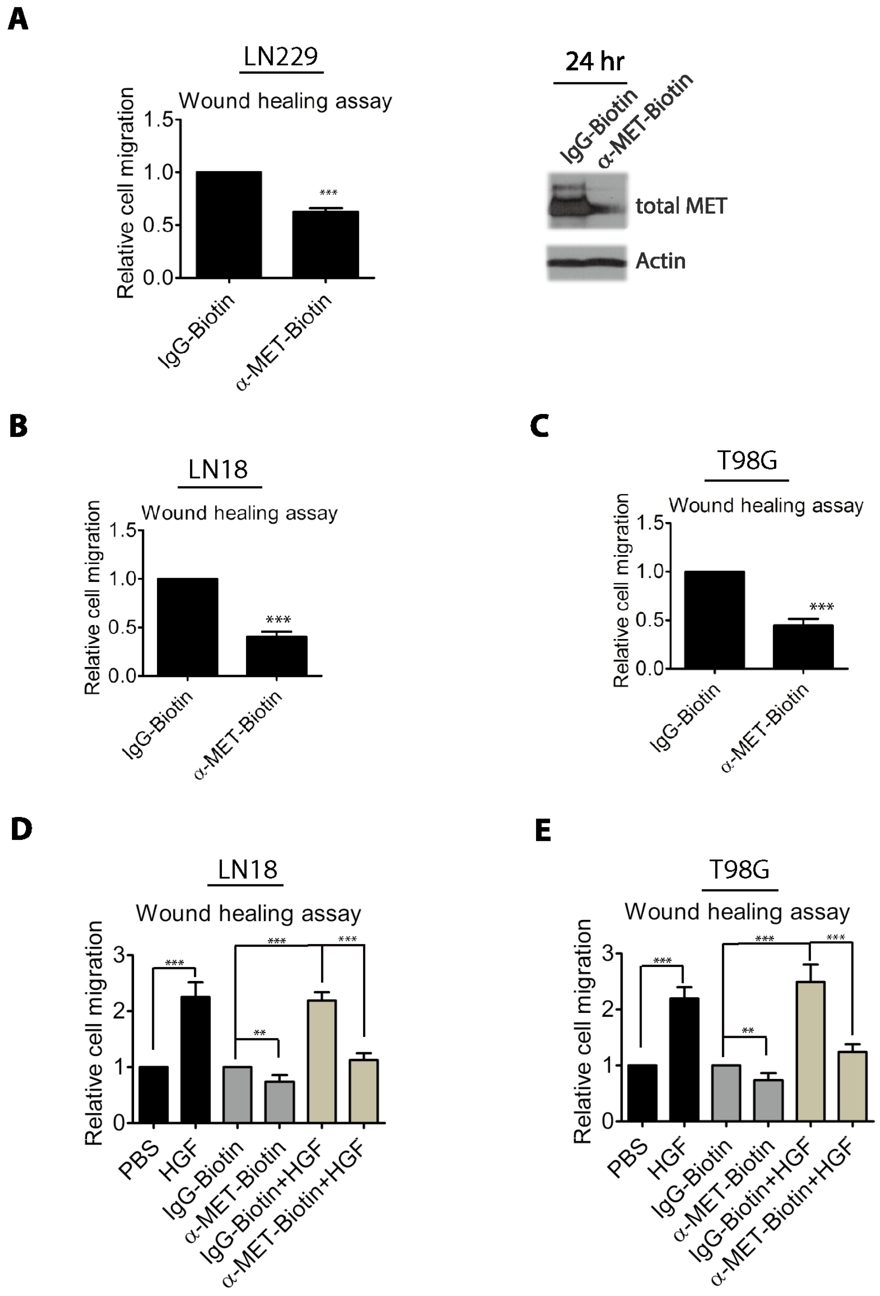

Suppl Figure 5. Anti-MET-biotin antibodies blocked cell migration in various cancer cells.

A. LN229 cells were seeded on the plate and cultured in complete medium for 16 hours. A sterile 20 µl tip was used to scrape the cells to make a wound. The cells were washed with warm PBS to remove floating cells and replaced with fresh complete medium. The biotin-conjugated anti-MET antibodies or biotin-conjugated control IgG, each at 2 µg/ml, were added to the cells for additional 24 hours. The live cells were photographed. The cells migrated into the wound area at the 24 hours were quantified from 6 independent fields and quantified. The means (with S.D.) from a duplicate set of samples were calculated. Statistical analysis was conducted (compared to IgG control) (statistical significance *** $p<0.001$). Right panel: Parallel samples were similarly

treated and subsequently lysed in SDS buffer and the total levels of MET protein and actin (loading control) were examined by Western blotting.

B. The same as A except LN18 cells were analyzed.

C. The same as A except T98G cells were analyzed.

D. LN18 cells were seeded on the plate for 16 hours and then serum-starved for additional 9 hours. A scrape wound was made on each plate as in panel A (time point 0 hour). To each plate, HGF (50 ng/ml) or PBS (control) was added; or control antibodies (IgG-Biotin, 2 μg/ml) or anti-MET antibodies (α-MET-Biotin, 2 μg/ml), either alone or together with HGF, were added to the media as indicated. Cells were further incubated for 24 hours and live cells were photographed. The cells migrated into the wound area at the 24 hour time point were quantified, and normalized to that when cells were treated with PBS control, and the means (with S.D.) from a duplicate set of samples were calculated and shown in panel C. Statistical analysis was conducted for each indicated pair (statistical significance *** $p<0.001$, ** $p<0.01$).

E. The same as in D except T98G cells were used.
